# Proteomic Investigation Uncovers Potential Targets and Target Sites of Pneumococcal Serine-Threonine Kinase StkP and Phosphatase PhpP

**DOI:** 10.3389/fmicb.2019.03101

**Published:** 2020-02-04

**Authors:** Claudia Hirschfeld, Alejandro Gómez-Mejia, Jürgen Bartel, Christian Hentschker, Manfred Rohde, Sandra Maaß, Sven Hammerschmidt, Dörte Becher

**Affiliations:** ^1^Department of Microbial Proteomics, Institute of Microbiology, University of Greifswald, Greifswald, Germany; ^2^Department of Molecular Genetics and Infection Biology, Interfaculty Institute for Genetics and Functional Genomics, University of Greifswald, Greifswald, Germany; ^3^Department of Functional Genomics, Interfaculty Institute for Genetics and Functional Genomics, University Medicine Greifswald, Greifswald, Germany; ^4^Central Facility for Microscopy, Helmholtz Centre for Infection Research, Braunschweig, Germany

**Keywords:** *Streptococcus pneumoniae*, Ser/Thr kinases, phosphatases, phosphoproteomics, mass spectrometry, label-free quantification, phosphopeptide enrichment, spectral library

## Abstract

Like eukaryotes, different bacterial species express one or more Ser/Thr kinases and phosphatases that operate in various signaling networks by catalyzing phosphorylation and dephosphorylation of proteins that can immediately regulate biochemical pathways by altering protein function. The human pathogen *Streptococcus pneumoniae* encodes a single Ser/Thr kinase-phosphatase couple known as StkP-PhpP, which has shown to be crucial in the regulation of cell wall synthesis and cell division. In this study, we applied proteomics to further understand the physiological role of pneumococcal PhpP and StkP with an emphasis on phosphorylation events on Ser and Thr residues. Therefore, the proteome of the non-encapsulated D39 strain (WT), a kinase (Δ*stkP*), and phosphatase mutant (Δ*phpP*) were compared in a mass spectrometry based label-free quantification experiment. Results show that a loss of function of PhpP causes an increased abundance of proteins in the phosphate uptake system Pst. Quantitative proteomic data demonstrated an effect of StkP and PhpP on the two-component systems ComDE, LiaRS, CiaRH, and VicRK. To obtain further information on the function, targets and target sites of PhpP and StkP we combined the advantages of phosphopeptide enrichment using titanium dioxide and spectral library based data evaluation for sensitive detection of changes in the phosphoproteome of the wild type and the mutant strains. According to the role of StkP in cell division we identified several proteins involved in cell wall synthesis and cell division that are apparently phosphorylated by StkP. Unlike StkP, the physiological function of the co-expressed PhpP is poorly understood. For the first time we were able to provide a list of previously unknown putative targets of PhpP. Under these new putative targets of PhpP are, among others, five proteins with direct involvement in cell division (DivIVA, GpsB) and peptidoglycan biosynthesis (MltG, MreC, MacP).

## Introduction

*Streptococcus pneumoniae* (pneumococcus) is a commensal bacterium of the human nasopharynx but has emerged as a serious opportunistic human pathogen that can colonize the human upper respiratory tract and can transmigrate into the major organs blood and the nervous system, causing several severe and invasive infections including pneumonia, otitis media, bacteremia and meningitis (Mitchell, 2000; Bogaert et al., 2004; Ramirez et al., 2015). The occurrence of invasive pneumococcal disease is increased in young children, in elderly, debilitated and immunosuppressed individuals (Welte et al., 2012; Song et al., 2013). According to the World Health Organization (WHO), in 2015 pneumococcal pneumonia was responsible of the death of approximately 920,136 children under the age of five. Actually, this corresponds roughly to 16% of the total deaths of children under 5 years old (World Health Organization, 2016). Although there are vaccines available against several pneumococcal serotypes, not all serotypes are covered by vaccination. In addition to that the emergence of antimicrobial resistance in bacterial infections has become a major public health concern worldwide. The pneumococcus overcomes not only the stress and starvation conditions of the ever-dynamic settings of bacterial habitats, but also the host defense mechanisms and antibiotic treatments (Koppe et al., 2012; Saleh et al., 2013; Kohler et al., 2016; Rabes et al., 2016). Additionally, *S. pneumoniae* is increasingly resistant to the most common clinically used drugs such as *beta*-lactam antibiotics and macrolides (Schulz and Hammerschmidt, 2013; Gladstone et al., 2019; Peyrani et al., 2019). Moreover, it can express a high number of specific virulence factors that raise pathogenicity by complex mechanisms (Bogaert et al., 2004; Kadioglu et al., 2008; Gamez and Hammerschmidt, 2012; Voss et al., 2012; Doran et al., 2016; Loughran et al., 2019; Subramanian et al., 2019). To ensure successful treatments in the future, it is vital to develop new antimicrobial drugs against pneumococci and their resistant strains, which are not based on the composition of the polysaccharide capsule but on other virulence factors like specific proteins, e.g., choline-binding proteins or lipoproteins (Pérez-Dorado et al., 2012; Kohler et al., 2016; Voß et al., 2018). Basic research of pneumococcal protein regulation is therefore required to decipher the molecular and cellular mechanisms that underlie pathogenesis and virulence.

In prokaryotic organisms’ phosphorylation/dephosphorylation cascades play a key role in protein regulation ensuring the adaptation of the cells to steadily changing environmental conditions. The most abundant signaling systems in bacteria including pneumococci are two-component regulatory systems consisting of a histidine kinase coupled to a cognate response regulator (Jung et al., 2012; Gómez-Mejia et al., 2017). However, previously published studies have demonstrated that eukaryotic-type Ser/Thr protein kinases (ESTKs) as well as Ser/Thr phosphatases (ESTPs) are present in a wide range of bacterial species and operate in parallel or overlapping signaling networks thereby constituting another important signaling mechanism for the regulation of different cellular functions (Shi et al., 2014; Wright and Ulijasz, 2014; Zhang et al., 2017; Janczarek et al., 2018). The pneumococcus presents only one gene encoding an ESTK, *stkP*, and its respective ESTP, *phpP*. The Ser/Thr kinase gene *stkP* is located downstream of the *phpP* gene and encodes the only annotated PASTA domain (penicillin-binding protein and Ser/Thr kinase associated domain) containing membrane-associated protein kinase StkP (Echenique et al., 2004; Nováková et al., 2005). However, the presence of another putative Ser/Thr phosphatase (SPD_1061) has also been reported (Agarwal et al., 2012) but the function and characteristics of the protein are not yet discovered. Furthermore, there are no sequence similarities to PhpP and conserved catalytic motifs (I-XI) that are typical for the eukaryotic-like PP2C family of phosphatases are missing (Shi, 2009).

The PhpP and StkP proteins are in the focus of this study. Both proteins appear to constitute a functional signaling couple *in vivo* and likely belong to the same complex (Osaki et al., 2009). StkP has been extensively investigated and its involvement in the regulation of different cellular processes has been reported. It is already known that StkP contributes to virulence and its presence is relevant for lung infection and bloodstream invasion as well (Echenique et al., 2004; Herbert et al., 2015; Piñas et al., 2018). Moreover, it regulates pilus expression and bacterial adherence (Echenique et al., 2004; Nováková et al., 2005; Herbert et al., 2015; Grangeasse, 2016). Pneumococcal StkP is also crucial for the resistance of *S. pneumoniae* to differing stress conditions and competence development. A transcriptome analysis showed that StkP affects the transcription of a set of genes encoding functions involved in cell wall metabolism, pyrimidine biosynthesis, DNA repair, iron uptake and oxidative stress response (Sasková et al., 2007). Furthermore, it was demonstrated that StkP localizes to the division sites and participates in the regulation of cell division. Pneumococci with *stkP* mutations revealed disrupted cell wall synthesis and displayed elongated morphologies with multiple, often delocalized, cell division septa. This led to the assumption that StkP modulates cell wall synthesis and cell division and thus contributes to the characteristic ovoid shape of *S. pneumoniae* (Beilharz et al., 2012; Fleurie et al., 2014b; Zucchini et al., 2018). Reflected to its role in cell division, several proteins involved in cell wall synthesis and cell division were found to be phosphorylated by StkP. The cell division proteins DivIVA (Nováková et al., 2010; Fleurie et al., 2012), MapZ (Nováková et al., 2010; Fleurie et al., 2014b; Holečková et al., 2014) and the phosphoglucosamine mutase GlmM (Nováková et al., 2005) were shown to be phosphorylated by StkP *in vitro* and *in vivo*. In addition, FtsZ (Giefing et al., 2010) and FtsA (Beilharz et al., 2012) and the cell wall biosynthesis enzyme MurC (Falk and Weisblum, 2013) are substrates of StkP *in vitro*; nonetheless, their phosphorylation by StkP *in vivo* has not yet been confirmed. Unlike StkP, the physiological function of the co-expressed PhpP, which is necessary for regulation of StkP activity, is poorly understood in pneumococci. In general, only a few cognate ESTPs have been studied in detail. However, several of them have been demonstrated to be essential for bacterial survival since it was possible to generate either ESTK or ESTK-ESTP double mutants but not ESTP mutants in *Streptococcus pyogenes*, *Streptococcus agalactiae*, or *Bacillus anthracis* (Jin and Pancholi, 2006; Alber, 2009; Burnside et al., 2010). Other studies of phosphatase knockout mutants showed that bacteria were viable and that ESTPs play a role in virulence, cell wall metabolism and cell segregation (Rajagopal et al., 2003; Beltramini et al., 2009; Banu et al., 2010; Burnside et al., 2010; Sajid et al., 2015). The first streptococcal ESTP mutant in *S. pyogenes* was described by Agarwal et al. (2011). Among others it was shown that the Ser/Thr phosphatase influences the expression of surface proteins and functions as an important regulator of group A streptococcal virulence. Subsequently, the first PhpP mutant in the encapsulated *S. pneumoniae* D39 strain was generated and characterized, thereby demonstrating that PhpP is not essential for pneumococcal growth or survival (Agarwal et al., 2012). Another successful construction of a Δ*phpP* mutant in the non-encapsulated *S. pneumoniae* Rx1 strain by Ulrych et al. (2016) led to the identification of a novel PhpP substrate, the putative RNA binding protein Jag. Moreover, the pneumococcal phosphatase PhpP is known to dephosphorylate the cognate kinase StkP. PhpP is a PP2C-type Mn^2+^-dependent enzyme, containing 11 conserved signature motifs. Mutations of the highly conserved PhpP residues D192 and D231, implicated in metal binding, completely suppressed PhpP activity *in vitro* (Bork et al., 1996; Nováková et al., 2005). PhpP is localized in the cytoplasm and often the GFP fused protein was found enriched in the midcell. However, it has been demonstrated that the localization of PhpP to cell division sites depends on the presence of active StkP (Beilharz et al., 2012; Ulrych et al., 2016). Since ESTKs participate in the regulation of cell division and peptidoglycan synthesis, it was furthermore shown that deletions of ESTPs in diverse bacteria also altered normal cell division and/or bacterial growth. For instance, deletion of the *Streptococcus mutans* phosphatase PppL, like its cognate kinase PknB, resulted in abnormal cell shapes and slower growth in comparison to the wild-type strain (Banu et al., 2010). A current approach toward the characterization of the pneumococcal phosphatase PhpP suggests that PhpP and StkP cooperatively regulate cell division of *S. pneumoniae*, moreover, the authors revealed a novel PhpP substrate, SPD_1849, a putative RNA binding protein Jag (SpoIIIJ-associated protein) (Ulrych et al., 2016).

To address the global impact and physiological relevance of StkP and PhpP we applied proteomics with an emphasis on protein phosphorylation events that are related to regulation of cell morphology, growth and cell division (Massidda et al., 2013). Thus, the non-encapsulated D39 strain (WT), the isogenic kinase (Δ*stkP*) and phosphatase mutants (Δ*phpP*) were analyzed in a label-free global proteome quantification experiment using GeLC-MS/MS. To obtain further information on the activity, targets and target sites of PhpP and StkP we combined the advantages of phosphopeptide enrichment using titanium dioxide and spectral library based data evaluation for an in-depth analysis of the phosphoproteome.

## Experimental Procedures

### Bacterial Growth

The construction and a table of *S. pneumoniae* strains used in this study can be found in the Supplementary Data Sheet S1. Pneumococci were grown on Columbia blood agar plates (Oxoid) for 6–8 h at 37°C in an atmosphere of 5% CO_2_. Bacteria were cultured onto a fresh blood agar plate containing the selective antibiotics kanamycin (50 μg/ml) and erythromycin (5 μg/ml) and incubated for approximately 8–9 h at 37°C and 5% CO_2_. Pneumococcal liquid cultures were prepared in modified RPMI 1640 medium (Schulz et al., 2014) and incubated at 37°C in a water bath without agitation in 50 ml conical tubes. For label-free quantification (LFQ) experiments RPMI 1640 medium without L-glutamine, phenol red (GE Healthcare Bio-Sciences) supplemented with 30.52 mM glucose, 2.05 mM glutamine, 0.65 mM uracil, 0.27 mM adenine, 1.1 mM glycine, 0.24 mM choline chloride, 1.7 mM NaH_2_PO_4_⋅H_2_O, 3.8 mM Na_2_HPO_4_, and 27 mM NaHCO_3_, 20 mM HEPES according to Schulz et al. (2014), was used. For SILAC-based proteome quantification, bacteria were cultivated in SILAC RPMI 1640 Flex Media, without glucose, phenol red, L-glutamine, L-lysine, L-arginine (Thermo Fisher Scientific), adding the listed supplements with additionally 11.11 mM glucose and heavy labeled arginine (Arg-10:HCl, 200 mg l^–1^) and lysine (Lys-8:HCl, 40 mg l^–1^) (Silantes). Liquid cultures were inoculated to starting optical densities (OD_600 nm_) between 0.05 and 0.06 and grown to exponential growth phase at OD_600 nm_ 0.3–0.35. In order to reach almost complete incorporation (five to six generation times) of heavy labeled amino acids in SILAC standards, two subsequent pre-cultures were grown before inoculating the main cultures. To verify the incorporation rate a MS analysis was executed as described in Hoyer et al. (2018). All experiments were carried out in three independent biological replicates.

### Field Emission Scanning Electron Microscopy (FESEM)

Samples for FESEM analysis were prepared according to Hoyer et al. (2018). Briefly, pneumococci were grown until exponential phase in RPMI *modi* medium and fixed with 5% formaldehyde (v/v) and 2% glutaraldehyde (v/v) in growth medium and kept at 7°C. The samples were washed twice with TE buffer (20 mM tris, 2 mM ethylene-diamine-tetra acetic acid (EDTA), pH 6.9). Bacterial cells were attached onto poly-L-lysine coated cover slips (12 mm in diameter) for 15 min, fixed with 1% (v/v) glutaraldehyde in TE buffer, washed twice with TE buffer and dehydrated with an increasing concentration of acetone [10, 30, 50, 70, 90, 100% (v/v)] on ice for 15 min each. Samples in 100% acetone were handled at room temperature and transferred one more time to 100% acetone. Afterward, samples were subjected to critical-point drying with liquid CO_2_ (CPD 030, Bal-Tec). Dried samples were mounted with carbon adhesive tape onto aluminum stubs and coated with an approximately 8 nm thick gold-palladium film by sputter coating (SCD 500 Bal-Tec) before examination in a field emission scanning electron microscope (Zeiss Merlin) using the Everhart-Thornley SE-detector alone or coupled to the Inlens SE-detector in a 75:25 ratio at an acceleration voltage of 5 kV.

### Embedding in LRWhite Resin and Transmission Electron Microscopy (TEM)

Sample preparation for TEM analysis was performed as described in Hoyer et al. (2018). Briefly, fixed bacteria were centrifuged and the resulting cell sediment was mixed with an equal volume of 1.75% (w/v) water agar. After solidification the agar was cut into small cubes and dehydrated with an increasing concentration of ethanol in 30 min [10, 30, 50% (v/v)] steps on ice. Samples were incubated in 70% ethanol with 2% uranyl acetate overnight. Samples were dehydrated with 90 and 100% ethanol. This step was repeated twice before the samples were infiltrated with one part 100% ethanol and one part LRWhite resin (London resin Company) overnight. Next, samples were infiltrated with one part 100% ethanol and two parts LRWhite resin for 24 h and subsequently infiltrated with pure LRWhite resin with two exchanges over 2 days. Then 1 μl starter was added to 10 ml LRWhite resin, stirred and resin was transferred into 0.5 ml gelatin capsules. After polymerization for 4 days at 50°C, ultrathin sections were cut with a diamond knife. Sections were counter-stained with 4% aqueous uranyl acetate (w/v) for 1 min. Samples were imaged using a Zeiss TEM 910 transmission electron microscope at an acceleration voltage of 80 kV at calibrated magnifications. Images were recorded digitally at calibrated magnifications with a Slow-Scan CCD-Camera (ProScan, 1024 × 1024) with ITEM-Software (Olympus Soft Imaging Solutions).

### Sample Preparation for Global Identification and Label-Free Quantification Using GeLC-MS/MS

For classical proteome analysis applying a label-free quantification strategy, pneumococci were harvested by centrifugation (4,500 × g, 10 min, 4°C) and washed three times with ice cold 1x PBS (18.25 mM NaCl, 931.90 mM KCl, 246.45 mM Na_2_HPO_4_, 1.42 M KH_2_PO_4_) supplemented with 1% (w/v) choline chloride and 1x cOmplete protease inhibitor cocktail (Roche) according to the manufacturer’s protocol. The bacterial sediments were suspended in 750 μl 50 mM tris-HCl buffer with 10 mM EDTA, pH 7.4. Bacterial cell disruption was performed by bead beating with a Precellys homogenizer (6,000 rpm, 9 × 30 s, 4°C). Glass beads and cell debris were removed by subsequent centrifugation steps (5,000 × g, 15 min, 4°C; 10,000 × g, 45 min, 4°C). After protein concentration determination with the Bradford assay (Bradford, 1976) using Roti-Nanoquant (Roth) according to the manufacturer’s protocol, proteins were pre-fractionated by SDS-PAGE and resulting gel lanes were cut into 10 pieces per sample (Bonn et al., 2014). After removal of salt and non-proteinogenous contaminants by incubation in a buffer containing 0.2 M ammonium bicarbonate in 30% (v/v) acetonitrile, gel-pieces were dried in a vacuum centrifuge and rehydrated with an aqueous solution of trypsin (2 ng ×μl^–1^). Proteins were then digested with trypsin overnight and the resulting peptides eluted in ultrapure water. Subsequently, samples were analyzed by mass spectrometry.

### Sample Preparation for Phosphoproteome Analysis

For phosphoproteome experiments, harvested bacteria were washed with 50 mM tris-HCl buffer, pH 7.4, supplemented with 5 mm C_3_H_7_O_6_PNa_2_, 5 mm Na_4_P_2_O_7_, 5 mm Na_3_VO_4_ and 10 mm NaF and disrupted as described for the GeLC-MS/MS proteome samples. Afterward the suspension was treated with 1 × Nuclease Mix (GE Healthcare) for 20 min at RT and with 1% (w/v) Octyl β-d-glucopyranoside (Sigma-Aldrich, 5 min, 4°C) before cell debris and glass beads were removed by two centrifugation steps (5,000 × g, 15 min, 4°C; 10,000 × g, 45 min, 4°C). The protein concentration was determined to ensure the precipitation of 5 mg protein for each sample. Methanol/chloroform precipitation was followed by sample digestion with Lys-C and trypsin and bead based phosphopeptide enrichment by titanium dioxide (TiO_2_) as described elsewhere (Olsen and Macek, 2009). Briefly, precipitated samples were resolved in denaturation buffer (6 M urea, 2 M thiourea in 10 mM tris-HCl, pH 8.0) and disulfide reduction as well as cysteine alkylation were performed. Samples were digested for 8 h with Lys-C, diluted and further digested for 12 h with trypsin. After sample acidification to pH 2.7 with trifluoroacetic acid (TFA) and centrifugation, each sample was repetitively incubated with TiO_2_ beads to collect nine enrichment fractions. Therefore, beads were suspended in a required volume (50 μl per 5 mg beads) of loading solution (30 mg/ml 2,3-dihydroxybenzoic acid (DHB), 80% (v/v) acetonitrile (ACN) in ASTM type 1 water) and incubated for 10 min at room temperature in an orbital shaker in the dark. Afterward, TiO_2_ beads were added to each sample. Following bead to peptide ratios were used for the enrichment: 5 mg TiO_2_ beads/1 mg protein for fraction 1, 3 mg TiO_2_ beads/1 mg protein for fraction 2, 1 mg TiO_2_ beads/1 mg protein for fraction 3. This procedure was repeated twice. After incubation for 15 min in an orbital shaker (in the dark, RT), the samples were centrifuged (10,000 × g, 2 min, RT) and the supernatant was discarded. The beads were washed twice with each wash solution [wash solution 1: 30% (v/v) ACN, 3% TFA (v/v); wash solution 2: 80% (v/v) ACN, 0.1% TFA (v/v)] and eluted three times (elution buffer: 40% (v/v) NH_4_OH, 60% (v/v) ACN, pH > 10.5) using C_8_-microcolumns (Thermo Fisher Scientific) packed with 3–4 × 1 mm^2^ pieces of 3 m Empore C_8_ material.

### Mass Spectrometric Analysis

All proteomic samples were analyzed by reversed phase liquid chromatography (LC) electrospray ionization (ESI) MS/MS using an LTQ Orbitrap Velos (Thermo Fisher Scientific) as described by Sokolov et al. (2019). In brief, in-house self-packed nano-LC columns (100 μm × 20 cm) containing reverse-phase C_18_ material (3.6 μm, Aeris, phenomenex) were used to perform LC with an Easy-nLC1000 system (Thermo Fisher Scientific). The peptides were loaded with solvent A [0.1% acetic acid (v/v)]. Subsequently, the peptides were eluted by a non-linear binary gradient of 80 min from 5 to 99% solvent B [0.1% acetic acid (v/v), 99.9% ACN (v/v)] in solvent A at a constant flow rate of 300 nl/min.

After injection of non-enriched proteomic samples into the mass spectrometer, a full scan was recorded in the Orbitrap (m/z 300–1,700) with a resolution of 30,000 at 400 m/z. The 20 most abundant precursor ions were consecutively isolated in the linear ion trap and fragmented via collision-induced dissociation (CID). Unassigned charge states as well as singly charged ions were rejected and the lock mass correction was enabled. Selected ions were fragmented with 35% normalized collision energy (NCE) and analyzed in data-dependent MS/MS in the linear trap quadrupole (LTQ).

During the measurement of the phosphopeptide-enriched samples a full scan with a resolution of 60,000 at 400 m/z was recorded in the Orbitrap (m/z 300–2,000). The 20 most abundant precursor ions were fragmented via CID and recorded in the LTQ analyzer. Lock mass correction, wideband activation and multistage activation at −97.98, −48.99, −32.70, and −24.49 Th were enabled.

### Database Search and Label-Free Quantification

For classical database search, spectra were searched separately for each strain with MaxQuant (1.6.1.0, Max Planck Institute of Biochemistry; Cox and Mann, 2008) and its implemented search engine Andromeda (Cox et al., 2011) against the *S. pneumoniae* D39 database from UniProt (2017) containing 1,918 proteins. Parameters for precursor mass deviation were set to 4.5 ppm and 0.5 Da for fragment mass tolerance. A false discovery rate (FDR) of 0.01 on protein, peptide and spectra level was applied as well as a minimum peptide length of seven amino acids and two unique peptides per protein. Full tryptic specificity with a maximum of two missed cleavage sites was applied. Variable modifications were oxidation on methionine (M) and N-terminal acetylation (Protein N-term). The label-free quantification option LFQ was activated (min. ratio count set to two) as well as the option “match between runs” for the bioreplicates of each strain. Furthermore, MaxQuant’s generic contamination list was included.

For shot gun proteomic data analyses the software Perseus 1.6.1.1 (Tyanova et al., 2016) was used. The data from MaxQuant output files were filtered for contaminations, reverse and only identified by site hits. The quantification of comparative proteome analysis of D39Δ wild-type and mutants was based on MaxQuant’s LFQ intensities (Cox et al., 2014). Proteins were considered for quantification if an LFQ value was present in all three biological replicates of each strain. Data were normalized over the median in Microsoft Excel. Statistical evaluation of quantified proteins was performed using RStudio (version 3.5.0) and the SAM (Significance analysis of microarrays; Tusher et al., 2001) script by Michael Seo^1^ with the implemented two-class unpaired test, which is analogous to a *t*-test between subjects. The input data tables can be found in Supplementary Data Sheet S2. Differentially expressed proteins with a minimum fold change of two and a *q* ≤ 0.01 were considered as significantly regulated. The results were visualized in Voronoi tree maps (Bernhardt et al., 2013) using the software Paver 2.0 (DECODON) as previously described in Hoyer et al. (2018).

### Construction of a Spectral Library and Spectral Library Search for Phosphoproteomics

**FIGURE 1:**
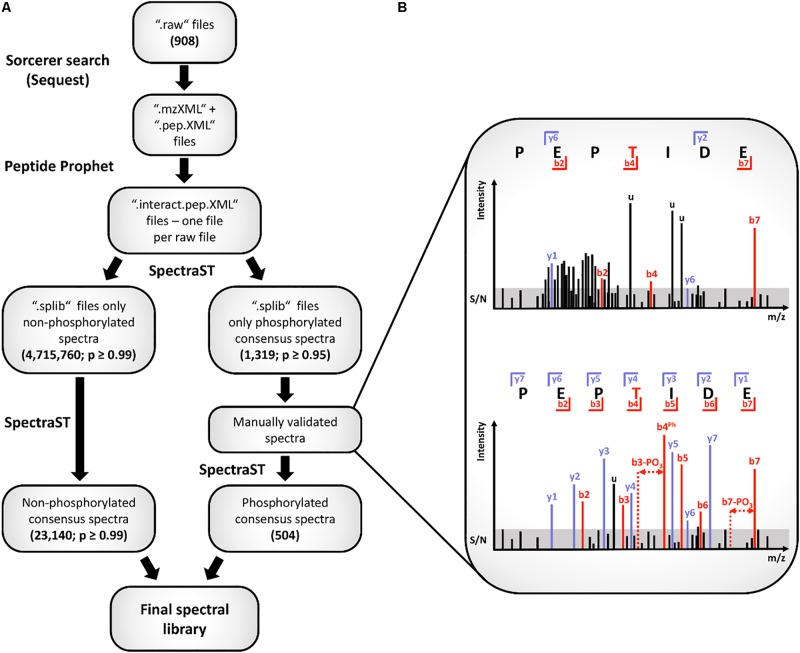
Workflow for spectral library creation. The particular data-processing steps of the spectral library generation are depicted in **(A)** of the figure. All spectra belonging to the phospho-library were validated manually. Illustration **(B)** represents a discarded (upper spectrum) and a kept spectrum (lower spectrum) according to the applied filter criteria. For unambiguous spectrum identification, five of the six listed criteria had to be fulfilled (see main text). In the upper spectrum, peak clusters, unassigned peaks (u) as well as identified peaks not sufficient above or even below the signal to noise ratio (S/N) are visible (b4, y6). Additionally, no fragment ion series, no fragments with neutral losses or the phosphorylation site are assigned. In contrast to that, the lower spectrum displays a complete fragment ion series, identification of the fragment including the phosphorylation site and fragments with a neutral loss of phosphoric acid. Further, the signals of assigned fragment peaks are above the S/N range as well as only a limited number of unassigned peaks is visible.

For generation of the combined spectral library 908 raw files of *S. pneumoniae* samples available from a defined set of experiments were considered (see Supplementary Data Sheet S3). First, all samples were searched against the *S. pneumoniae* D39 database from UniProt (1,918 proteins, 2017) with Sorcerer – SEQUEST 4.0.4 (SageN). For the respective parameter files, precursor mass tolerance was set to 10 ppm and fragment mass tolerance was set to 1.0005 Da (CID). Full tryptic specificity with a maximum of two missed cleavage sites was considered as well as variable modifications like oxidation on methionine, carbamidomethylation on cysteine and phosphorylation on serine (S), threonine (T), and tyrosine (Y). For SILAC samples an additional search with static modifications on lysine [Lys C^13^N^15^ (K), 8.014199 amu] and arginine [Arg C^13^N^15^ (R), 10.008269 amu] was done. For further processing of the MS/MS data in. mzXML and. pep. XML format from the Sorcerer output the Trans Proteomic Pipeline (TPP, 5.1.0-rc1 Sysygy, 2017) was applied. Spectral library creation was performed according to Schubert et al. (2015) with slight modifications. Briefly, spectra and their particular identifications retrieved from all files were linked together and entries from all datasets were combined to interact. pep. XML files (InteractParser) (Figure 1A). Afterward the PeptideProphet algorithm was applied to calculate the false discovery rate (FDR) of the merged searches on peptide level. The following settings were used: minimum peptide sequence length: seven amino acids, use accurate mass binning, using: ppm, use decoy hits to pin down the negative distribution, ignore charge states: 1+ and higher than 4+. Then spectra containing no phosphorylated amino acids were imported to build a raw spectral library if they had a calculated probability of at least 0.99. In parallel a raw library containing spectra with S/T/Y phosphorylated amino acids and with a probability of at least 0.95 was built. Within this step, spectra assigned to the same peptide ion identification were merged and a single, representative “consensus” spectrum for a particular “peptide species” was generated. Consensus spectra of phosphorylated peptides were then subjected to manual validation and only spectra conformed five out of six quality criteria were considered for the library. Following filter criteria were used for manual review of spectra: (1) Identified fragments should not be within clusters of masses. (2) No more than three dominating unassigned peaks (u) should be in a spectrum. (3) Fragment intensities should be sufficiently above the signal to noise threshold (S/N). (4) Peptide fragments should be detected with and without phosphorylation (−PO^3^). (5) At least three fragments should be detected post-phosphosite. (6) The phosphosite itself (Ph) should be identified within a spectrum. The illustration in Figure 1B exemplifies the listed filter criteria for manual validation. The whole procedure of data processing and validation was repeated to build two raw libraries with the spectra of the SILAC samples which contain the heavy labeled amino acids lysine and arginine. Overall, four raw libraries were built: 1st: including non-phosphorylated spectra; 2nd: including non-phosphorylated spectra of SILAC samples; 3rd: including phosphorylated spectra; 4th: including phosphorylated spectra of SILAC samples. Afterward the four raw libraries were combined and filtered for contaminations and reverse hits. The final spectral library for phosphorylation-centered searches was then extended with the same number of spectra as decoy hits generated by a random mass shift of the precursor and shuffling the peptide sequence (Yen et al., 2011). Finally, phospho-enriched samples of D39Δ*cps*, D39Δ*cps*Δ*stkP*, and D39Δ*cps*Δ*phpP* were searched against the combined spectral library using SpectraST. Search results were combined to interact.pep. xml files and filtered for probability of at least 0.95.

### Experimental Design and Statistical Rationale

For global protein identification and label-free quantification using GeLC-MS/MS analysis pneumococci of WT, Δ*stkP*, and Δ*phpP* were cultivated in three independent biological replicates on different days using freshly prepared RPMI *modi* medium. All samples were prepared in parallel prior to LC-MS/MS analysis. For the identification proteins must have been detected with at least two unique peptides. Proteins were considered for quantification if LFQ values were available for three out of three biological replicates of each strain. Statistical significance was assessed by a two-class unpaired test reported above. For spectral library construction, proteome samples of *S. pneumoniae* were prepared after cultivation under diverse growth conditions in parts with biological and technical replicates, generating samples for whole cell extracts and cell culture supernatant (908 raw files, origin is provided in Supplementary Data Sheet S3). Raw spectral libraries for non-phosphorylated peptides considered hits with a probability of at least 0.99 (PeptideProphet). Spectra containing S/T/Y phosphorylated amino acids with a probability of at least 0.95 were added to the library when they passed the additional manual validation described before. For data analysis based on spectral libraries, three independent biological replicates of WT and the mutants Δ*phpP* and Δ*stkP* were then subjected to SpectraST search (81 raw files). SpectraST search results were filtered afterward for a probability ≥ 0.95. For phosphopeptide identification only peptides that were found in three out of three biological replicates in each strain were considered.

### Data Availability

The MS proteomics data, the spectral library and the MaxQuant outputs have been deposited to the ProteomeXchange Consortium via the PRIDE partner repository (Vizcaíno et al., 2016) with the dataset identifier PXD015268. Annotated MS/MS spectra of the spectral library search results can be viewed with the help of the identified peptide sequence provided in the Supplementary Data Sheet S4 and the freely available MS-Viewer tool, accessible through the Protein Prospector suite of software at the following URL: http://prospector2.ucsf.edu/prospector/cgi-bin/msform.cgi?form=msviewer, with the search key: t3hpidqwm8.

## Results and Discussion

### Protein Identification and Label-Free Proteomic Analysis of Kinase and Phosphatase Mutant

**FIGURE 2:**
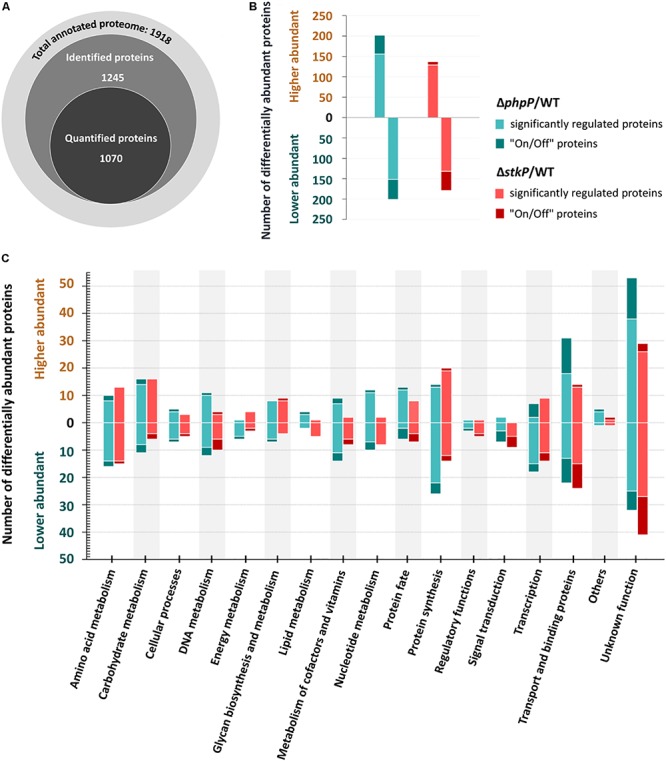
Global protein identification and label-free quantification results. An overview of identified and quantified protein numbers within the GeLC-MS/MS approach is shown in **(A)**. The highest number of significant changes in the proteome was found in Δ*phpP*/WT (156 higher abundant proteins and 47 “on” proteins, 152 lower abundant proteins and 49 “off” proteins). The Δ*stkP*/WT analysis revealed 129 proteins with a higher abundance, 9 “on” proteins, 132 less abundant proteins and 47 “off” proteins) as summarized in **(B)**. **(C)** Sums up the absolute numbers of proteins belonging to the respective functional class and significantly quantified in at three out of three replicates of the mutants/WT. Only proteins with significantly (*q* ≤ 0.01) higher ratios (≥2×) are displayed.

In order to characterize the impact of StkP and PhpP on cellular physiology we performed a mass-spectrometry based label-free quantitative (LFQ) approach to determine changes on proteome level in pneumococci deficient for StkP or PhpP. The global proteome analysis conducted in this study resulted in the high-confidence detection of 1245 pneumococcal proteins, representing 65% of the annotated proteome of *S. pneumoniae* strain D39. Out of the identified proteins, 1,070 proteins met the criteria for quantification, reflecting a quantification efficiency of 86% (Figure 2A). Within the LFQ analysis the mutants have been compared to the non-encapsulated wild type strain D39Δ*cps*. A significantly higher number of protein abundance changes (fold change ≥ 2, *q* < 0.01) was found in the phosphatase mutant (Δ*phpP*/WT – 403 regulated proteins) compared to the kinase mutant (Δ*stkP*/WT – 313 regulated proteins) (Figure 2B). Overall, 156 higher abundant and 152 lower abundant proteins could be identified in Δ*phpP*/WT. In addition to that, 46 proteins with at least two unique peptides in three out of three replicates occur exclusively in Δ*phpP* but not in the WT (“On” proteins). On the other hand, there are 49 proteins identified only in the WT but in none of the replicates of the phosphatase mutant (“Off” proteins). In the Δ*stkP*/WT comparison, fewer proteins with altered abundance have been detected, i.e., 129 higher abundant and 132 lower abundant proteins. Furthermore, only seven additional “On” proteins have been identified in the kinase mutant but 45 “Off” proteins are present in the WT but not in Δ*stkP*. All identified unique peptides and the corresponding “On/Off” protein identification results can be found in Supplementary Data Sheet S5. For further insights regarding impacts of StkP and PhpP on particular biological processes in the pneumococcus, all 1918 predicted proteins of *S. pneumoniae* D39 were classified into functional protein groups according to TIGRFAM (Haft et al., 2001) or KEGG-based (Kanehisa and Goto, 2000) annotation (Supplementary Data Sheet S6). Proteins with significantly altered abundances were observed in almost every functional group in both mutant strains (Figure 2C). Notable changes in the proteome of the kinase mutant were especially observed in proteins belonging to the categories “Amino acid metabolism,” “Energy metabolism,” “Regulatory functions,” “Signal transduction,” and “Transcription.” In the proteome of the phosphatase mutant, proteins of the mentioned categories were also found to be remarkably changed in abundance. Additionally, changes in the metabolism of DNA, nucleotides, carbohydrates, cofactors and vitamins as well as in the categories “Transport and binding proteins” and “Glycan biosynthesis and metabolism” have been observed in the proteome of the phosphatase mutant. A detailed overview of the label-free quantification data is provided in Supplementary Data Sheet S2.

**FIGURE 3:**
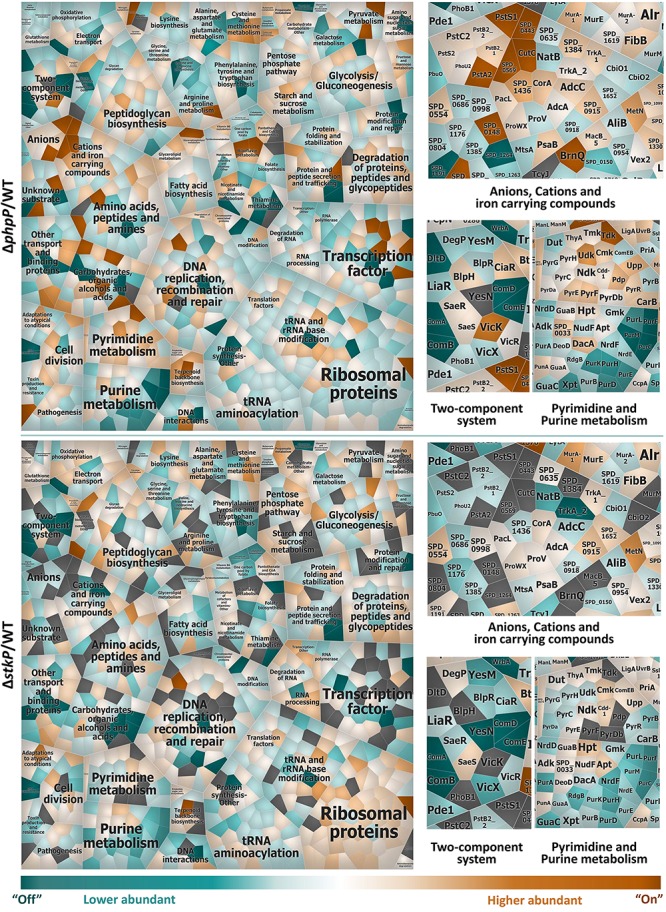
Comparison of protein abundances Δ*phpP*/WT and Δ*stkP*/WT. Voronoi treemap of the quantified proteome (1,070 proteins of the 1,918 theoretical proteins) of pneumococcal phosphatase and kinase mutant in comparison to WT assigned to specific functional sub roles. Within the treemap all tiles symbolize single proteins which are colored according to their abundance in a divergent color gradient. Turquoise cells indicate proteins that are less abundant or “Off” in the mutant in comparison to the WT whereas higher abundant or “On” proteins are illustrated in orange. Gray fields represent proteins that could not be quantified in the compared strains. Proteins involved in the same biological process are clustered together (complete Voronoi treemap collection labeled with main and sub roles and single proteins see Supplementary Image S1). For a deeper insight into changes of protein abundances in the cluster of anions, cations and iron carrying compounds, in two-component systems as well as in pyrimidine and purine metabolism, the protein level is depicted next to the overall treemaps.

For a more detailed view on differences in the proteome pattern of *stkP* and *phpP* mutants all regulated proteins were clustered by a more specific functional annotation in a Voronoi treemap (Figure 3). Among others, noticeable changes in the protein abundance in the mutant strains in comparison to the WT have been observed especially in the cluster of anions, cations and iron carrying compounds, in two-component systems as well as in pyrimidine and purine metabolism.

### Loss of Function of PhpP Increased Abundance of Proteins, e.g., in Phosphate Uptake System Pst

Within the group of transport and binding proteins, the ABC transporter complex PstSCAB, which is involved in phosphate import, presented a higher abundance in the phosphatase mutant but was less abundant in the kinase mutant when compared to the WT, respectively (Figure 3 and Supplementary Data Sheet S2). All proteins included in the *pst*2 operon, namely PhoU2, PstA2, PstB2_1, PstB2_2, PstBC2, and PstS2 could be identified and quantified in Δ*phpP*. The phosphate import ATP-binding protein PstB2_1 (ATPase subunit 1) is significantly more abundant in Δ*phpP*/WT (FC 2.3) and the membrane channel protein PstA2, a putative phosphate ABC transporter, was identified only in the phosphatase mutant but neither in the WT nor in the kinase mutant. The phosphate import ATP-binding protein PstB2_2 (ATPase subunit 2) (FC 1.5) and the putative phosphate ABC transporter proteins PstC2 (FC 1.8) and PstS2 (FC 1.6) were also slightly more abundant in Δ*phpP*/WT. The phosphate-specific transport system accessory protein PhoU2 (FC 1.2) did not present any noticeable change in abundance. In the kinase mutant the putative phosphate-binding ABC transporter protein PstS2 was significantly less abundant in comparison to the WT (FC -2.3). PhoU2 (FC -1.5), PstB2_1 (FC -1.4), and PstB2_2 (FC -1.5) were slightly less abundant in Δ*stkP*/WT, too. Unlike other bacterial species, for example *E. coli*, *B. subtilis*, or *C. crescentus*, that contain only one Pst transporter, the pneumococcus encodes two evolutionarily separated phosphate ABC pump transporters, Pst1 and Pst2 in adaptation to different Pi concentrations in the human host (Lanie et al., 2007; Moreno-Letelier et al., 2011; Zheng et al., 2016). The *pst*1-*pho*U1 and *pst*2-*pho*U2 operons are completely separated in the pneumococcal chromosome. In a previous study it was shown, that the Pst2 transporter is constitutively expressed under high and low Pi conditions. *Pst*1 level increased under low Pi concentrations (Zheng et al., 2016). The phosphate-binding ABC transporter protein PstS1 of the pneumococcal inorganic phosphate uptake system 1 was identified exclusively in the phosphatase mutant and not in the WT or the kinase mutant. Other components of the *pst*1 operon were not identified in any of the strains. From these results it can be hypothesized, that especially PhpP affects phosphate uptake system Pst2 and probably also Pst1. Since PhpP is responsible for the dephosphorylation of phosphorylated StkP and its targets, it could be speculated that the missing dephosphorylation of StkP could effect the homoestatic phospohorylation level in the cell. Consequently cells may react by increasing abundance of the Pst system. However, this would need further experimental investigation.

Another hint, that indicates an influence of the PhpP on the overall phosphate uptake in the pneumococcal cell is the exclusive identification of the Na/Pi-cotransporter II-related protein SPD_0443 in the Δ*phpP* mutant.

Furthermore, proteins, that are not directly involved in the phosphate homeostasis but in the regulation of the intracellular concentration of other cations were also affected: In the adjacent cluster of cations and iron carrying compounds, the sodium/hydrogen exchange family protein SPD_0569 and the copper homeostasis protein CutC were identified only in Δ*phpP* but not in Δ*stkP* and WT. Additionally the magnesium transporter CorA, the zink ABC transporter AdcC, the potassium uptake protein TrkA (FC 2.03) and the cation-transporting ATPase SPD_1436 (FC 5.44) were significantly higher abundant in the phosphatase mutant in comparison to the WT, but not significantly changed in abundance in the kinase mutant. The iron-compound binding ABC transporter SPD_0915 (Δ*phpP*/WT FC 3.04; Δ*stkP*/WT FC 3.14) was significantly higher abundant in both mutants.

### Significant Changes in Protein Abundance in Purine and Pyrimidine Metabolism in *ΔphpP* and *ΔstkP* Mutants

Purine and pyrimidine nucleotides are major energy carriers for cellular processes, subunits of nucleic acids, precursors for the synthesis of nucleotide cofactors or are used for activation of precursors in polysaccharide and lipid synthesis (Kilstrup et al., 2005; Turnbough and Switzer, 2008). ESTKs and ESTKPs are important for the accurate balance of purine nucleotide pools and the regulation of purine biosynthesis (Rajagopal et al., 2005). Our data show that the adenylosuccinate synthetase PurA, which catalyzes the first committed step in the biosynthesis of adenosine monophosphate (AMP) from inosine monophosphate (IMP), was significantly less abundant in both mutants compared to the WT (Δ*phpP*/WT FC -2.6; Δ*stkP*/WT FC -7.4). Other proteins involved in the *de novo* purine biosynthesis (PurC, PurF, PurM) were “off” in the phosphatase mutant and less abundant in Δ*stkP*/WT (PurC FC -18.5; PurF FC -11.5; PurM FC -4.5) (Figure 3 and Supplementary Tables S2, S5). The N5-carboxyaminoimidazole ribonucleotide mutase PurE was significantly less abundant in the phosphatase mutant (FC -16.2). Among others, further proteins belonging to the IMP *de novo* biosynthesis (PurB, PurD, PurH, PurK, PurL) were less abundant in Δ*phpP*/WT (PurB FC -1.89; PurD FC -10.73; PurH FC -36.06; PurK FC -24.61; PurL -10.72) and in Δ*stkP*/WT (PurB FC -1.37; PurD FC -4.48; PurH FC -7.94; PurK FC -2.85; PurL -6.25). In contrast, the ribose-phosphate pyrophosphokinase SPD_0033, that plays a crucial role in the biosynthesis of the central metabolite phospho-*alpha*-D-ribosyl-1-pyrophosphate, was significantly more abundant in the phosphatase mutant (FC 2.9), but not significantly altered in abundance in the kinase mutant compared to the WT. The ribonucleoside-diphosphatase reductases NrdD, NrdE and NrdF, necessary for providing the precursors for DNA synthesis, were significantly less abundant in both mutants (Δ*phpP*/WT NrdD FC -3.74, NrdE FC -2.19, NrdF FC -2.19; Δ*stkP*/WT NrdD FC -2.99, NrdE FC -2.57, NrdF FC -7.34) likewise the guanosine monophosphate (GMP) reductase GuaC (Δ*phpP*/WT, FC -2.29; Δ*stkP*/WT FC -5.69) that catalyzes the irreversible NADPH-dependent deamination of GMP to IMP. The diadenylate cyclase DacA, part of the cyclic-AMP biosynthetic process, was significantly more abundant in the phosphatase mutant (FC 3.3), but not notably changed in Δ*stkP*/WT (FC -1.2).

A transcriptomic study indicated that a deletion mutant of an ESTK in *Staphylococcus aureus* affected the expression of genes belonging to regulons that are involved in purine and also in pyrimidine biosynthesis (Donat et al., 2009). As a result of our proteome analysis of the deletion mutants Δ*phpP* and Δ*stkP*, we observed significant changes in protein abundances in the functional cluster of pyrimidine metabolism especially in the phosphatase mutant. The orotate phosphoribosyltransferase PurE and the orotidine 5′-phosphate decarboxylase PyrF, both part of the uridine monophosphate (UMP) *de novo* biosynthetic pathway, are significantly more abundant in Δ*phpP*/WT (FC PyrE 2.81; FC PyrF 3.55). Other significantly higher abundant proteins in Δ*phpP*/WT involved in pyrimidine metabolism are: The uracil phosphoribosyl transferase Upp (FC 4.18), the pyrimidine-nucleoside phosphorylase Pdp (FC 4.07), the uridine kinase Udk (FC 8.4), the carbamoyl-phosphate synthase CarB (FC 2.49), the bifunctional protein PyrR (FC 2.38) and the cytidylate kinase Cmk (FC 2.53). Furthermore, the thymidine kinase Tdk was identified in all replicates of Δ*phpP* but not at all in the WT. In the kinase mutant these proteins were not notably changed in their abundance. Interestingly, two other proteins involved in the UMP *de novo* biosynthesis (PyrDa and PyrB), that were not changed in the phosphatase mutant, are significantly less abundant in Δ*stkP*/WT (FC PyrDa -2.58; FC PyrB -3.51).

### StkP and PhpP Interact With Two-Component Systems

It is reported for a number of prokaryotes that eukaryotic-like serine/threonine kinases and phosphatases crosstalk with two-component systems (Lin et al., 2009; Ulijasz et al., 2009; Didier et al., 2010; Burnside and Rajagopal, 2012). The results of our comparative proteome analysis of the deletion mutants Δ*phpP* and Δ*stkP* revealed variations of protein abundances in the pneumococcal ComCDE and ComAB systems (Figure 3), both with critical functions for pneumococcal competence and fracticide under specific environmental conditions (Gómez-Mejia et al., 2017). The response regulator ComE was significantly less abundant in Δ*phpP*/WT (FC -231) and Δ*stkP*/WT (FC -298.87). ComD, the putative sensor histidine kinase, was not detected in both mutants but in the WT. Furthermore, the competence factor transporting ATP-binding/permease protein ComA and the competence factor transport protein ComB, that were identified in each bioreplicate of the WT with more than 15 unique peptides, were not identified at all in the kinase mutant and in the phosphatase mutant. An influence of the deletion of *phpP* and *stkP* was also observed in the LiaRS two-component system. LiaRS is also involved in the competence process by responding to peptidoglycan cleavage by LytA, CbpD, and LytC murein hydrolases (Eldholm et al., 2010; Gómez-Mejia et al., 2017). The choline binding protein CbpD was reliable identified in the WT but not in the mutants Δ*phpP* and Δ*stkP*. The autolysin/N-acetylmuramoyl-L-alanine amidase LytA was significantly lower in both mutants in comparison to the WT (Δ*phpP*/WT, FC -2.96; Δ*stkP*/WT FC -2.91). Moreover, the DNA-binding response regulator LiaR was significantly less abundant in Δ*phpP*/WT and Δ*stkP*/WT. The cognate sensor histidine kinase LiaS was only quantified in Δ*phpP* and WT, but no appreciable changes in abundance were noticed. In case of the CiaRH (competence induction and altered cefotaxime susceptibility) system, a contrarily regulation in Δ*phpP*/WT and Δ*stkP*/WT was noticed. The DNA-binding response regulator CiaR was significantly more abundant in the phosphatase mutant (FC 2.97), but significantly less abundant in the kinase mutant in comparison to the WT (FC -2.95). CiaH, the corresponding sensor histidine kinase, was not identified in all strains. In another study it was shown, that CiaR causes an upregulation of the virulence factor HtrA (DegP) (Stevens et al., 2011). In contrast to that, within our proteomic data we found the serine protease DegP significantly lower abundant in Δ*phpP*/WT (FC -2.41) and slightly higher abundant in Δ*stkP*/WT (FC 1.41). Another pneumococcal two-component system, that is involved in the regulation of competence, is VicRK (TCS02, WalRK). The VicX ancillary protein encoded in the *vicRK* operon is shown to have a significantly lower abundance in the kinase mutant (FC -6.71) and was less abundant in Δ*phpP*/WT, too (FC -1.92). While there are no differences in the abundance of the sensory box sensor histidine kinase VicK in Δ*stkP*/WT, this protein is significantly more abundant in the phosphatase mutant (FC 44.03). The DNA-binding response regulator VicR is only slightly more abundant in the phosphatase mutant (FC 1.08) and less abundant in the kinase mutant (FC -1.5) in comparison to the WT. The essentiality of VicR in the pneumococcus is due to its positive regulation of a gene encoding a putative peptidoglycan hydrolase (PcsB, SPD_2043) that plays a critical role in cell wall biosynthesis and cell division. PcsB is known to interact with the cell division complex FtsE/FtsX (Sham et al., 2011) and was found in all three strains in our analysis. In the Δ*php*P mutant, PcsB was more abundant (FC 1.88), while no changes were seen in the Δ*stkP* strain. The cell division proteins FtsE (ATP-binding) and FtsX were significantly less abundant in the kinase mutant Δ*stkP* (FC FtsE -3.69; FC FtsX -4.77). No significant changes were detected in the abundance of FtsE or FtsX in the Δ*phpP* mutant.

### Role of StkP and PhpP in Growth, Cell Division, and Septum Formation

**FIGURE 4:**
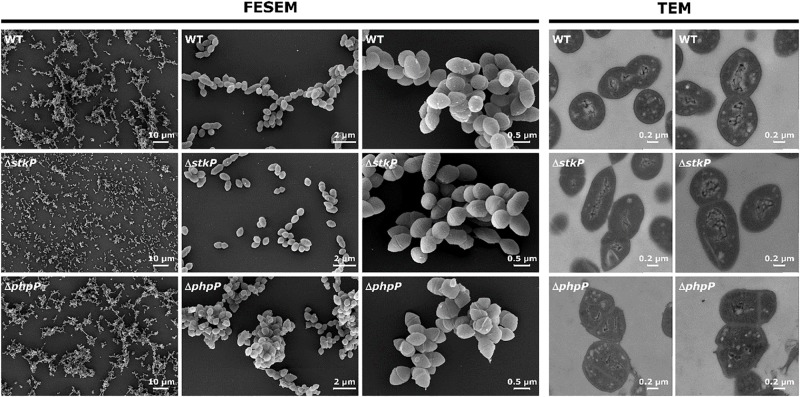
Microscopic analysis of WT, Δ*stkP*, and Δ*phpP* morphology. The morphological characterization of all strains by field emission scanning electron microscopy (FESEM) and transmission electron microscopy (TEM) pointed out that cell division and cell separation were abnormal in both mutants.

Eukaryotic-type kinases have been implicated in regulating cell division, correct septum progression and closure and thus bacterial growth in the pneumococcus and other pathogens (Beltramini et al., 2009; Agarwal et al., 2011; Beilharz et al., 2012; Fleurie et al., 2014b). As bacterial cell growth and division are closely linked, we first characterized the strains by growth in the chemically defined RPMI 1640 medium (Supplementary Data Sheet S1). The growth of WT and Δ*stkP* was almost comparable, whereas Δ*phpP* showed diminished growth and reached a lower final OD. This result goes along with the quantitative data from the label-free proteome analysis, where ribosomal proteins in Δ*phpP* are less abundant compared to the WT. On the contrary, ribosomal proteins in the Δ*stkP* strain were more abundant, most probably due to the faster growth of the kinase mutant in the early exponential phase (first 60 min of growth). Within the label-free quantification data we could not see notable significant alterations in the abundance of proteins in the functional clusters of cell division and peptidoglycan biosynthesis. Nevertheless, we analyzed the mutants and the WT by field emission scanning and transmission electron microscopy (FESEM, TEM). The electron microscopic analysis of pneumococcal cell morphology and cell division revealed remarkable differences in chain formation, cell separation, and cell division septa morphology between WT, Δ*phpP*, and Δ*stkP* (Figure 4). While cells of Δ*stkP* were forming fewer chains in comparison to the WT and were more separated, pneumococci of Δ*phpP* were forming aggregates. The cells of Δ*stkP* seemed to be perturbed in the progression of the septa during the cell division, often displaying elongated morphologies. The cell morphology of Δ*phpP* was unusual as well. Cells were forming multiple asymmetric septa and were not properly separated. Moreover, pictures derived from TEM analysis of Δ*phpP* revealed the DNA (light structure) to be delocalized and not as dense as in WT cells. Interestingly, the observed phenotypes of Δ*phpP* and Δ*stkP* mutants are not similar compared to those of earlier generated ESTP and ESTK mutants described (Agarwal et al., 2012; Ulrych et al., 2016). In the studies published by Ulrych et al. and Agarwal et al. strains were cultivated in complex media such as THY (Todd Hewitt broth with yeast extract) or C + Y (casitone and yeast) medium. Within this study all investigations were performed after culturing pneumococci in the chemically-defined and supplemented cell culture medium RPMI-1640. It has already been described that the medium used for cultivation can greatly influence the outcome of a physiological study in pneumococci (Schulz et al., 2014; Hoyer et al., 2018). Growth defects and morphological changes undetected in complex media are uncovered in CDM. Hence, we speculate that different phenotypic effects as well as proteomic responses are strain- but also cultivation media dependent. Still, a phenotype similar to that observed in this study was described by Agarwal et al. (2011) for an STP deletion mutant in *S. pyogenes*, also showing multiple asymmetric and parallel septa formation comparable to the observations presented in this study. Nevertheless, the results emphasize that PhpP and StkP participate cooperatively in the regulation of cell division in *S. pneumoniae*. Since no notable changes in the proteome pattern of Δ*stkP* and Δ*phpP* regarding cell division were detected, it strengthens the assumption that phosphorylation of proteins involved in this process causes the strong phenotypical effects.

### Comparative Phosphoproteomic Analysis of *ΔstkP*, *ΔphpP*, and WT

Our LFQ results suggest that PhpP and StkP are involved in the regulation of pneumococcal morphogenesis by modulating proteins belonging to cell division and associated processes like peptidoglycan biosynthesis by balancing the phosphorylation and dephosphorylation level of target proteins. Previously identified target substrates of StkP *in vivo* are DivIVA, GlmM, PpaC, MurC, MapZ, and StkP itself (Osaki et al., 2009; Nováková et al., 2010; Falk and Weisblum, 2013; Fleurie et al., 2014b; Hammond et al., 2019). Nevertheless, most of the target sites of StkP remain to be identified. However, the role of PhpP is not well determined. In order to be able to correlate the phenotypic observations to protein phosphorylation patterns, we performed a gel-free phosphopeptide enrichment with TiO_2_ followed by MS/MS measurement and spectral library based data evaluation.

**FIGURE 5:**
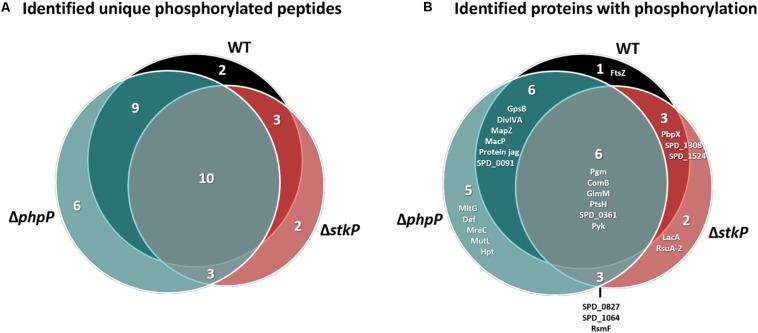
Phosphopeptide and phosphoprotein identifications in WT and mutant strains. **(A)** Number of phosphopeptides identified in the WT, Δ*phpP*, and Δ*stkP* mutant and overlap of identifications between the different investigated strains. **(B)** Overlap of phosphorylated proteins between mutants and WT. Only phosphorylations at serine, threonine, and tyrosine are considered.

In particular, it was of major interest to find possible new targets/target sites of StkP and especially PhpP. For our analysis, we considered only phosphorylated peptides that were found in three out of three biological replicates in each strain. Thus, we identified 24 unique phosphopeptides in the WT derived from 16 different proteins, 18 unique phosphopeptides in Δ*stkP* (14 proteins) and 28 unique phosphopeptides in Δ*phpP* (21 proteins). Among the identified phosphopeptides, 26 phosphorylation sites were determined in the WT: 11 on serine, 14 on threonine and one on tyrosine; in Δ*stkP* we found 20 phosphorylation sites: 11 on serine, 7 on threonine and 2 on tyrosine and in Δ*phpP* we detected 29 phosphorylation sites: 14 on serine, 14 on threonine and one on tyrosine. Overall, 10 phosphopeptides could be identified commonly in every strain (Figure 5). These peptides are derived from 6 proteins, that are involved in the carbohydrate metabolism (phosphoglucomutase Pgm, phosphoglucosamine mutase GlmM, pyruvate kinase Pyk), signal transduction (competence factor transport protein ComB), transport and binding (phosphocarrier protein PtsH) and one protein with unknown function (SPD_0361). A detailed overview of all identifications regarding phosphorylation is provided in Supplementary Data Sheet S4.

### Identification of Previously Observed StkP Targets and Detection of Putative New Target Substrates

To pinpoint targets of StkP the data were filtered for phosphorylated peptides that were identified in the WT in every replicate but in none of the replicates of the kinase mutant. Since the kinase StkP is disrupted in the mutant, its targets should not be phosphorylated compared to the WT. Yet we identified six phosphoylation sites within seven unique peptides belonging to 6 distinct proteins that can be considered with utmost probability (marked with ^∗∗∗^ in Table 1) to be targets of StkP (Table 1). Applying our MS based phosphoproteome approach we could support the assumption of other studies that the cell division proteins DivIVA and MapZ are targets of the StkP. Within the analysis of the phosphopeptide-enriched samples, a phosphorylation site on Thr201 of DivIVA was identified. This phosphorylation site of DivIVA was already detected in an earlier study focussing on the investigation of the phosphoproteome of *S. pneumoniae* (Sun et al., 2010). Further studies of the pneumococcus revealed that phosphorylation of proteins on threonine is StkP dependent (Nováková et al., 2010; Fleurie et al., 2012). Additionally, our results indicate that the prokaryotic tubulin homolog FtsZ, which interacts with MapZ and participates in the formation of the division septum, is phosphorylated by StkP. A phosphorylation site on Thr356 of FtsZ was detected in all replicates of the WT but in none of the Δ*stkP* mutant strain. Moreover we observed proteins of unknown function, SPD_1849 and SPD_0091, which can be considered as putative StkP targets. A phosphoylation site within the endolytic murein transglycosylase MltG (Thr155) identified in two out of three WT bioreplicates was not found in the Δ*stkP* mutant. Therefore MltG could be a new and notable (marked with ^∗∗^ in Table 1) putative target of StkP. Another phosphorylated protein that was identified in this study is MacP (SPD_0876). MacP is a membrane-anchored cofactor of the penicillin-binding protein PBP2a and thus involved in peptidoglycan synthesis in pneumococci (Fenton et al., 2018). A phosphoproteome study by Sun et al. (2010) led to the identification of a phosphorylation site on Thr32 in the at that time unknown protein SPD_0876. In Fenton et al. (2018) reported the identification of MacP as a substrate of StkP. Moreover the phosphorylation at residue Thr32 was confirmed by applying *in vitro* approaches including antiphospho-threonine immunoblots. In this study phosphorylation in MacP was detected *in vivo*. Interestingly, the phosphorylation was detected on Thr56. Fifty-five peptide spectrum matches (PSMs) of the phosphorylated peptide were found in the WT and 43 PSM counts were assigned to the corresponding unphosphorylated peptide (Supplementary Data Sheet S7). Within the LFQ data, the MacP protein was significantly higher in abundance in the kinase mutant in comparison to the WT. According to that, it can be speculated that the inability of protein activation by the inactive StkP leads to the accumulation of the protein in the mutant. Besides we identified a phosphorylation on Thr12 in the phosphocarrier protein PtsH in the WT not occurring in the kinase mutant, although serine phosphorylation in PtsH was detected in the mutant and in the WT but on different positions. Still the loss of the threonine phosphorylation in the *stkP* deletion mutant refers to a possible interaction of StkP and PtsH by phosphorylation.

**TABLE 1:** Putative targets and target sites of pneumococcal kinase StkP identified in this study.

**Significance**	**Locus ID**	**Peptides inclusive phosphosites**	**Protein description**	**Biological process**
***	SPD_1040	DFHVVAE**T_12_**GIHAR	Phosphocarrier protein HPr	Carbohydrates, organic alcohols and acids
***	SPD_1474	EVVSEVLGEPIPAPIEEEPIDM**T_201_**R	Cell division protein DivIVA	Cell division
***	SPD_1479	HFDMAE**T_356_**VELPK	Cell division protein FtsZ	Cell division
***	SPD_0342	EEFVE**T_78_**QSLDDLIQEMR	Mid-cell-anchored protein MapZ	Cell division
***	SPD_0342	KEEFVE**T_78_**QSLDDLIQEMR	Mid-cell-anchored protein MapZ	Cell division
***	SPD_0876	DHGFSQE**T_56_**LK	Membrane protein MacP	Peptidoglycan biosynthesis
***	SPD_1849	TVSEE**T_89_**VDLGHVVDAIK	Protein jag (SpoIIIJ-associated protein), putative	Unknown function
***	SPD_0091	EIVHLGLEDNDFDNDINPLET**T_116_**GAYLSPK	UPF0176 protein SPD	Unknown function
**	SPD_1346	KAEQAGPETPTPA**T_155_**ETVDIIR	Endolytic murein transglycosylase MltG	Peptidoglycan biosynthesis

Phosphorylated peptides that were identified in the WT in every replicate but in none of the replicates of the kinase mutant were considered as highly significant (***) putative target substrates of StkP. If the phosphorylation site was identified in two out of the bioreplicates of the WT but not in the kinase mutant, the protein was also considered to be a significant putative target of StkP (**). The phosphorylated amino acid is indicated as bold letter.

### Phosphoproteome Analysis Uncovered Previously Unknown Putative Targets of PhpP

PhpP negatively controls the level of protein phosphorylation in *S. pneumoniae* by direct dephosphorylation of target proteins and by dephosphorylation of the cognate kinase StkP (Ulrych et al., 2016). For the identification of putative PhpP targets, our focus was on phosphorylation sites that were found in at least two out of three bioreplicates in Δ*phpP* (Supplementary Data Sheet S7). Phosphorylation sites that were not detected in the WT, but in all replicates of the phosphatase mutant, were considered as highly significant (marked with ^∗∗∗^ in Table 2) putative target substrates of PhpP (Table 2). Proteins, that fulfill these criteria, were the DNA mismatch repair protein MutL (Ser119) and the hypoxanthine phosphoribosyltransferase Hpt (Thr107). Due to the fact that the target substrates of PhpP can also be found phosphorylated in the WT, we analyzed those proteins as well. To provide more confidence, we included the counts of peptide spectrum matches (PSMs) per phosphorylated peptide and PSMs of the corresponding unphosphorylated peptide in our analysis. Phosphopeptides with a higher number of PSMs/phosphopeptide in Δ*phpP* in comparison to the WT and less or comparable PSM counts of the unphosphorylated peptide in Δ*phpP* are listed as notable (marked with ^∗∗^ in Table 2) putative targets. Under these new putative targets of PhpP are four proteins with direct involvement in cell division (MapZ, DivIVA) and peptidoglycan biosynthesis (MltG, MreC). This observation may link the abnormal cell morphology of the Δ*phpP* pneumococci to the phosphorylation status of critical cell division proteins. Moreover, MapZ and DivIVA are also targets of StkP. These results again support the assumption that PhpP and StkP are acting as a functional signaling couple as previously suggested (Osaki et al., 2009) and participate in the process of cell division and morphogenesis. In addition to that it can be indicated that SPD_1849, the putative protein Jag (SpoIIIJ-associated protein), is a target of PhpP. Indeed, two phosphorylation sites, at Thr126 and Thr89, were identified in Δ*phpP* at SPD_1849. The peptide including the phosphorylation on Thr126 was identified with only 1 PSM in only one WT replicate and with 28 PSMs in the phosphatase mutant in two out of three bioreplicates. The unphosphorylated form of this peptide was not identified in both strains. The peptide with the phosphorylation site Thr89 counted 13 PSMs in the WT and 15 PSMs in the mutant. Interestingly this phosphorylation site was already described in another non-proteomic study (Ulrych et al., 2016). Other proteins that were integrated in the list of significant target substrates (marked with ^∗∗^ in Table 2) of PhpP are: the phosphoglucomutase Pgm, the competence factor transport protein ComB and the NOL1/NOP2/sun family protein RsmF. Another slightly significant [(marked with ^∗^ in Table 2) – more PSM counts of the unphosphorylated peptide have been detected, see Table 2 and Supplementary Data Sheet S7] putative target of PhpP is, among others, the cell cycle protein GpsB. For the DivIVA paralog GpsB it was shown that it is required for StkP localization and activity and therefore for StkP-dependent phosphorylation of DivIVA. Still, there was no phosphorylation of GpsB itself described (Fleurie et al., 2014a). Nevertheless, in this study phosphorylation sites in GpsB were detected on Thr79 and Ser107. While Thr79 was exclusively identified with two PSMs in the phosphatase mutant (one PSM for the unphosphorylated peptide), the phosphorylation site on Ser107 was identified with at least 15 PSMs/phosphopeptide in the WT (10 PSMs/unphosphorylated peptide) and six PSMs/phosphopeptide in Δ*phpP* (one PSM/unphosphorylated peptide). This result gives a strong hint that phosphorylation might occur in GpsB itself as well. According to our observations, GpsB seems to be dephosphorylated by PhpP, but is not directly phosphorylated by StkP because in the StkP deficient mutant the phosphorylation site in GpsB on Ser107 was identified in two out of three biological replicates with four PSM counts of the phosphorylated peptide (Supplementary Data Sheet S7). For *Bacillus subtilis* for example, it is known that GpsB is phosphorylated on Thr75 by the kinase PrkC (Pompeo et al., 2015). However, further experimental assays are necessary to confirm the putative phosphorylation site or sites in pneumococcal GpsB. Furthermore, a recent study reported that GpsB functions as an adapter for multiple cell wall enzymes in *B. subtilis, Listeria monocytogenes*, and also *S. pneumoniae*. Thus an interaction with the cell shape-determining protein MreC was confirmed in the pneumococcus (Cleverley et al., 2019). Within our mass spectrometry based phosphoproteome analysis we provide evidence, that MreC is phosphorylated in the pneumococcus and its dephosphorylation is executed by PhpP. Three phosphorylation sites were detected in MreC in the WT and the phosphatase mutant: Ser141, Ser155, and Ser157. Two PSM counts of the phosphorylated peptide were found in the WT and even nine in Δ*phpP*. The unphosphorylated peptide was not detected in both strains. The membrane spanning cell wall synthetic factor MreC is poorly characterized. Interestingly the detected phosphorylation sites are located in the periplasmic like region according to the structure model of MreC (Lovering and Strynadka, 2007). Whether the phosphorylation of MreC is reliable and possible in that region has to be clarified. Moreover, we could not identify MreC as a target substrate of StkP. In future experiments it has to be elucidated which enzyme or what kind of interaction could be responsible for the phosphorylation in MreC. Other phosphorylation sites or further proteins with identified phosphorylation sites in the phosphatase mutant like LacA or AtpA cannot be completely rejected from the putative PhpP target list, but have to be considered as not significantly prominent in our data set. Nevertheless, the presented highly confident phosphorylation sites can hint to yet unknown dephosphorylation targets of the pneumococcal PhpP and thus provides the basis for a deeper insight into the regulatory mechanisms of this phosphatase. However, phosphorylation is dynamic and can change rapidly (Macek et al., 2009; Calder et al., 2016). For sure, not all phosphorylation sites could be detected within this MS based approach focused on an extension of available phosphoproteomic data for *S. pneumoniae* with an emphasis on the StkP/PhpP couple. A more detailed and complete description of proteins targeted for phosphorylation and dephosphorylation events can be obtained combining different techniques, for example structural analyses of possible target proteins, phenotypic characterizations of different gene mutants for possible targets or well established phosphoactivity assays.

**TABLE 2:** Putative targets and target sites of pneumococcal phosphatase PhpP identified in this study.

**Significance**	**Locus ID**	**Peptides inclusive phosphosites**	**Protein description**	**Biological process**
***	SPD_0165	GEALPSIASVSVLTLLTAVDGA**S_119_**HGTK	DNA mismatch repair protein MutL	DNA replication, recombination and repair
***	SPD_0012	HVLFVEDIIDTGQ**T_107_**LK	Hypoxanthine phosphoribosyltransferase Hpt	Purine metabolism
**	SPD_1390	LGVLATPAVAYLVETEGASAGVMISA**S_101_**HNPALDNGIK	Phosphoglucosamine mutase GlmM	Amino sugar and nucleotide sugar metabolism
*	SPD_1390	LGVLATPAVAYLVETEGA**S_93_**AGV**M**ISASHNPALDNGIK	Phosphoglucosamine mutase GlmM	Amino sugar and nucleotide sugar metabolism
–	SPD_1390	LGVLATPAVAYLVETEGASAGVMI**S_99_**ASHNPALDNGIK	Phosphoglucosamine mutase GlmM	Amino sugar and nucleotide sugar metabolism
**	SPD_1474	EVVSEVLGEPIPAPIEEEPID**MT_201_**R	Cell division protein DivIVA	Cell division
*	SPD_1474	EVVSEVLGEPIPAPIEEEPIDM**T_201_**R	Cell division protein DivIVA	Cell division
**	SPD_0342	KEEFVE**T_78_**QSLDDLIQEMR	Mid-cell-anchored protein MapZ	Cell division
*	SPD_0342	EEFVE**T_78_**QSLDDLIQEMR	Mid-cell-anchored protein MapZ	Cell division
*	SPD_0339	KPKPSPVQAEPLEAAI**T_79_**SS**M**TNFDILK	Cell cycle protein GpsB	Cell division
*	SPD_0339	QILDN**S_107_**DF	Cell cycle protein GpsB	Cell division
**	SPD_2045	GA**S_141_**EN**M**LAIANGGLIG**S_155_**V**S_157_**K	Cell shape-determining protein MreC	Peptidoglycan biosynthesis
**	SPD_1346	KAEQAGPETPTPA**T_155_**ETVDIIR	Endolytic murein transglycosylase MltG	Peptidoglycan biosynthesis
*	SPD_0876	DHGFSQE**T_56_**LK	Membrane protein MacP	Peptidoglycan biosynthesis
**	SPD_0050	ASTSQQNETIASQNAAASQTQAEIGNLI**S_182_**Q**T_184_**EAK	Competence factor transport protein ComB	Two-component system
**	SPD_1326	HLN**C**FAGIMVTA**S_144_**HNPAPFNGYK	Phosphogluco/mannomutase family protein Pgm	Glycolysis/Gluconeogenesis
*	SPD_1326	HLN**C**FAGIMV**T_142_**ASHNPAPFNGYK	Phosphogluco/mannomutase family protein Pgm	Glycolysis/Gluconeogenesis
*	SPD_0790	AICEE**T_238_**GNGHVQLFAK	Pyruvate kinase Pyk	Glycolysis/Gluconeogenesis
	SPD_0790	FNF**S_58_**HGDHQEQGER	Pyruvate kinase Pyk	Glycolysis/Gluconeogenesis
*	SPD_1040	**S_46_**IMGV**M**SLGVGQGADVTISAEGADADDAIAAISETMEK	Phosphocarrier protein PtsH	Carbohydrates, organic alcohols and acids
*	SPD_1040	SI**M**GV**MS_52_**LGVGQGADVTISAEGADADDAIAAISET**M**EK	Phosphocarrier protein PtsH	Carbohydrates, organic alcohols and acids
*	SPD_1285	IVSHSVQDAALGEGEGCL**S_132_**VDR	Peptide deformylase Def	Protein modification and repair
–	SPD_1053	EVVKDFLEKENFHLVDV**T_33_**AEGQDFVDVTLAVAAEVNK	Galactose-6-phosphate isomerase subunit LacA	Galactose metabolism
–	SPD_1337	ELEAF**T_396_**K	ATP synthase subunit alpha	Oxidative phosphorylation
–	SPD_1064	LVFAVDVG**T_115_**NQLAWK	Hemolysin-like protein, putative	Pathogenesis
–	SPD_1524	LQIVSHTLEPNQQLP**T_36_**VR	Transcriptional regulator, GntR family protein	Transcription factor
–	SPD_0592	ELPTV**M**DLLPSDIQ**S_92_**DK	Pseudouridine synthase	tRNA and rRNA base modification
**	SPD_1233	FEPSFALGLALKP**S_371_**QVEQ**S_376_**VEIGQEAFVK	NOL1/NOP2/sun family protein	Unknown function
**	SPD_1849	HAS**T_126_**ILEETGHIEILNELQIEEAMR	Protein jag (SpoIIIJ-associated protein), putative	Unknown function
**	SPD_1849	TVSEE**T_89_**VDLGHVVDAIK	Protein jag (SpoIIIJ-associated protein), putative	Unknown function
*	SPD_0361	LLTESGFVTNEALQEC**T_109_**K	Transcriptional regulator, putative	Unknown function
–	SPD_1308	**S_218_**VAQIALAWSLAEGFLPLPK	Oxidoreductase, aldo/keto reductase family protein	Unknown function
–	SPD_0091	EIVHLGLEDNDFDNDINPLET**T_116_**GAYLSPK	UPF0176 protein SPD	Unknown function

Phosphorylation sites that were not detected in the WT, but in all replicates of the phosphatase mutant were considered as highly significant (***) putative target substrates of PhpP. Phosphopeptides with a higher number of PSMs/phosphopeptide in ΔphpP in comparison to the WT and less or comparable PSM counts of the unphosphorylated peptide in ΔphpP are listed as notable (**) putative targets, too. Less significantly, but still mentionable putative target substrates of PhpP have more PSM counts for the identified unphosphorylated peptides and not remarkable more counts of PSMs for the phosphorylated peptides in ΔphpP in comparison to the WT (*). Proteins detected with phosphorylated peptides in ΔphpP are still in the putative PhpP target list, but were not significantly prominent in our data set (–). The phosphorylated amino acid is indicated as bold letter.

## Conclusion

### Role of Eukaryotic-Type Ser/Thr Protein Kinase StkP and Cognate Phosphatase PhpP in Pneumococci

**FIGURE 6:**
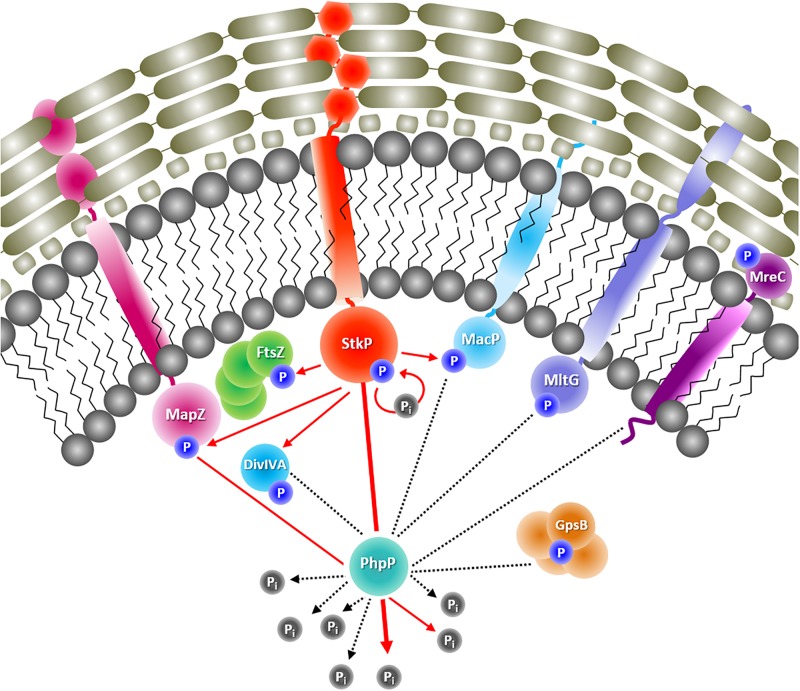
Identified putative target proteins of StkP and PhpP involved in pneumococcal cell division and peptidoglycan synthesis. Phosphorylated proteins participating in pneumococcal morphogenesis are presented. It is known, that StkP autophosphorylates and PhpP dephosphorylates StkP. StkP also phosphorylates the cell division proteins FtsZ and DivIVA as well as MapZ and MacP, both involved in peptidoglycan synthesis. For MapZ it was shown, that it is dephosphorylated by PhpP (red arrows = known interactions from literature, partially validated in this study). Data from this study also provide hints for an interaction of PhpP with the cell division proteins DivIVA and GpsB and moreover with MacP, MltG, and MreC, which are also participating in peptidoglycan synthesis (black dashed lines = putative interactions derived from this study).

This study provides a complex and comprehensive protein repository of high proteome coverage of *S. pneumoniae* D39 including identification of serine/threonine/tyrosine phosphorylation, which will facilitate further investigations of this important human pathogen. The global proteome analysis of the presented pneumococcal kinase and phosphatase mutants demonstrated once more the essential role of ESTKs and ESTPs in the protein regulation of the pneumococcus. Within the LFQ approach, proteins with significantly altered abundances were detected in diverse functional groups in both mutants. Most prominent changes in the proteome of the *stkP* mutant were observed in metabolic processes such as “Amino acid metabolism” and also in pathways regulating genetic and environmental information processing like “Transcription” and “Signal transduction”. Notable significant changes in the metabolism of DNA, nucleotides, carbohydrates, cofactors and vitamins as well as in the categories “Transport and binding proteins” and “Glycan biosynthesis and metabolism” have been additionally observed in the proteome of the phosphatase mutant. More detailed, the obtained data showed that the loss of function of the phosphatase PhpP resulted in an increased abundance of proteins attached to the pneumococcal phosphate uptake system Pst. Furthermore, the acquired data revealed an influence of the activity loss of either PhpP or StkP on proteins belonging to the pneumococcal two component-signaling systems ComDE, CiaRH, LiaRS and VicRK. Changes in protein abundances in the purine and pyrimidine metabolism in Δ*phpP* and Δ*stkP* mutants also refer to the more promiscuous mode of action of serine/threonine kinases in comparison to histidine kinases in two-component systems and resulting pleiotropic effects. Recent studies of the StkP/PhpP couple showed that both play an essential role in cell growth, cell division and separation. Consistently, growth analyses and the phenotypic characterization of the mutants by electron-microscopy pointed out that Δ*phpP* and Δ*stkP* have obviously different growth characteristics and abnormal cell division and cell separation. Due to the fact that the morphological effects could not be explained by changes in protein abundances on a global scale, the in depth analysis of the phosphoproteome was indispensable to provide further information of PhpP and StkP and their influence in cell division and peptidoglycan synthesis by modulation proteins participating in this mechanisms (Figure 6). However, studies of bacterial phosphoproteomes applying classical database search approaches to enriched proteomic samples usually reported less than 150 phosphorylated proteins. Previous investigations showed that the application of spectral libraries results in an improved sensitivity and reproducibility (Ge and Shan, 2011). Indeed, slight modifications within the workflow and the use of our recently build combined spectral library, including phosphoenriched samples and classical proteome samples of a high number of different growth and cultivation conditions, highly enhanced reliable and reproducible identification of phosphorylated proteins in this study (data not shown). Moreover, already known targets and target sites of StkP and PhpP, identified and investigated in other studies using different techniques, have been detected as a proof of principle applying the presented MS based phosphoproteome approach. Especially the list of putative targets of PhpP has been extended remarkably in this study. Nonetheless, some proteins that have already been described as StkP targets like MurC and GlmM were not detected in this study. In case of GlmM, which was identified *in vitro* and *in vivo* as StkP target in previous studies (Nováková et al., 2005, 2010), it was excluded from the StkP target list generated in this study. Applying the presented MS-based in-depth phosphoproteome analysis, phosphorylated peptides of GlmM were identified in all replicates of the Δ*stkP* mutant. For this reason, it can be speculated that another, unknown and not yet annotated Ser/Thr kinase in D39 might be responsible for the phosphorylation of further proteins. Still, the obtained data provide an extensive source of information for further investigations of new possible targets and target sites of StkP and PhpP. Moreover the constructed and validated spectral library with an emphasis on phosphorylation including a large number of phospho peptides (504) have been deposited to the ProteomeXchange Consortium via the PRIDE partner repository (Vizcaíno et al., 2016) with the dataset identifier PXD015268 and can thus be applied and extended by the scientific community.

## Data Availability Statement

The datasets generated for this study can be found in the MS proteomics data, the spectral library and the MaxQuant outputs have been deposited to the ProteomeXchange Consortium via the PRIDE partner repository (Vizcaíno et al., 2016) with the dataset identifier PXD015268. Annotated MS/MS spectra of the spectral library search results can be viewed with the help of the identified peptide sequence provided in Supplementary Data Sheet S4 and the freely available MS-Viewer tool, accessible through the Protein Prospector suite of software at the following URL: http://prospector2.ucsf.edu/prospector/cgi-bin/msform.cgi?form=msviewer, with the search key: t3hpidqwm8.

## Author Contributions

DB directed and supervised the project. DB and SH initiated the study, provided the resources, and revised the manuscript. CHi performed research, analyzed the data, and wrote the manuscript. SM analyzed the data and revised the manuscript. AG-M contributed to the mutant construction. JB assisted with the spectral library construction. CHe provided raw files for the spectral-library construction. MR performed the electron microscopy. All authors read and approved the final manuscript.

## Conflict of Interest

The authors declare that the research was conducted in the absence of any commercial or financial relationships that could be construed as a potential conflict of interest.

## Footnotes

1 https://github.com/MikeJSeo/SAM
