# Supplementary material for: Proteomic Investigation Uncovers Potential Targets and Target Sites of Pneumococcal Serine-Threonine Kinase StkP and Phosphatase PhpP
Source: Front Microbiol. 2020 Feb 4;10:3101. doi: 10.3389/fmicb.2019.03101 (PMC7011611; doi:10.3389/fmicb.2019.03101)
Supplement: Supplementary file 1 [file Data_Sheet_1.pdf]

Experimental procedures

Mutant construction

For the construction of the *stkP* mutant in D39Δ*cps* (Rennemeier et al., 2007), a DNA fragment consisting of the *S. pneumoniae* TIGR4 *stkP* gene (*sp_1732*) and its up- and down-stream flanking regions were amplified by PCR from genomic DNA using primers stkPStartForw and stkPEndRev (Table S1). The purified PCR product was cloned into pGEM-T Easy. The resulting plasmid pGEM-T Easy-*stkP* (pGstkP1) was transformed in chemically competent *E. coli* DH5α cells and positive mutants were selected using blue/white screening and verified by PCR. pGstkP1 was subsequently cleaved with *Bgl*II and purified. In parallel, an erythromycin resistance gene cassette containing *loxP*-sites was cloned into pGEM-T Easy, cleaved with *Nco*I and *Sac*I and cloned into pET28c. Afterwards, the antibiotic-cassette was amplified with the primers pGEM_rev and lox66_forw and cloned into the *Xcm*I cleaved vector pGXT. The erythromycin-cassette was excised with *Bam*HI and ligated into the *Bgl*II cleaved pGstkP1 plasmid. The recombinant plasmid pGstkP1::*erm* harboring the disrupted *stkP* gene was transformed into *E. coli* DH5α. Positive colonies were selected on ampicillin and finally on erythromycin. The D39Δ*cps*Δ*stkP* mutant was obtained after transformation of D39Δ*cps* with pGstkP1::*erm*. Transformants were verified for the correct integration of the antibiotic resistance cassette into the *stkP* gene region.
For the construction of the pneumococcal phosphatase mutant in D39Δ*cps* WT strain, a DNA fragment consisting of the *spd*_1543 gene was amplified by PCR from chromosomal DNA using the specific primers PhpPStartForw and PhpPEndRev. The purified PCR product was cloned into pGXT and *E. coli* DH5α chemically competent cells were transformed with the resulting plasmid. The recombinant plasmid pGXT harboring the desired DNA insert was purified and used as template for an inverse PCR reaction with primer pair PhpPStartForw and PhpPEndRev. The deleted gene sequence was replaced by an erythromycin gene cassette, amplified by PCR from vector pET28c-Erm^R-loxP using primer ermforw and ermrev. The final recombinant plasmid was used to transform and mutagenize pneumococci. The deletion of *phpP* was verified by PCR.

Table S1.: Bacterial strains, plasmids, and PCR primers used in this study.

Strain, plasmid, or primer	Resistance or sequence	Source or reference
***E.coli* DH5α**	None	Betheseda Research Labs, Gaithersburg, USA
S. pneumoniae		
TIGR4	None	(Tettelin et al., 2001)
D39Δ*cps*	Km^R	(Rennemeier et al., 2007)
D39Δ*cps stkP*::erm^R	Km^R, Erm^R	This study
D39Δ*cps*Δ*phpP*::erm^R	Km^R, Erm^R	This study
**Plasmids**		
pGEM®-T Easy	Amp^R	Promega
pGXT	Amp^R	(Chen et al., 2009)
pET28TEV	Km^R	(Saleh et al., 2013)
pGEM®-T Easy-ChS-Erm	Amp^R, Erm^R	This study
pGXT Erm^R	Amp^R, Erm^R	This study
pGEM®-T Easy-*stkP*	Amp^R	This study
pGEM®-T Easy Δ*stkP*	Amp^R	This study
pET28c Erm^R-loxP	Amp^R, Erm^R	This study
pGEM®-T Easy Erm^R- loxP	Amp^R, Erm^R	This study
pGEM-T Easy *stkP*::ErmR	Amp^R, Erm^R	This study
pGXT-*phpP*	Amp^R	This study
pGXT*phpP*::Erm^R	Amp^R, Erm^R	This study
**Primer**		
stkPStartForw	5’-CGCAAGATATCGGATTAGGAAGG-3’	
stkPEndRev	5’-TCATAATATCACGGACCGCATTGG-3’	
stkPStartRev	5’-AAGCGCATGCCTTGCCGATTTGGATCATTC-3’	
stkPEndForw	5’-GCGCGCATGCATCTACAAACCTAAAACAAC-3’	
ChS-Erm-F	5’-GCGCGCGCGGATCCCTGCAGTTGGCTTACCGTTCGTATAGC-3’	
ChS-Erm-R	5’-GCGCGCGGATCCAAGCTTTACCGTTCGTATAATGTATGCTATACGAAGTTATCCCAGTCT TTCGACTGAGCC-3’	
lox66_Erm_forw	5’-TACCGTTCGTATAGCATACATTATACGAAGTTATACGGTTCGTGTTCGTGCTG-3’	
lox71_Erm_rev	5’-ACCGTTCGTATAATGTATGCTATACGAAGTTATGTAGGCGCTAGGGACCTC-3’	
lox66_forw	5’-GGGGGGGGGGGGGCCCGGGTACCGTTCGTATAGCATACAT-3’	
lox71_rev	5’-GACAAAAAAAAAAAGATATCTACCGTTCGTATAATGTATGC-3’	
pGEM_rev	5’-GACAAAAAAAAAAAGATATCCCATATGGTCGACCTGCAG-3’	
PhpPStartForw	5´-GGGAAAACAGCCCATATAGC-3’	
PhpPEndRev	5´-TCCCCGTCATAAGGGATATG-3’	
PhpPEndForw	5´-AAGGAAGCTTCATTACGGTTGCCCTTGTTT-3’	
PhpPStartRev	5´-AAGGGCTAGCTTGTTCGTTTCTGACCAACAT-3’	
ermforw	5´-GCGCGCCTGCAGACGGTTCGTGTTCGTGCTG-3’	
ermrev	5´-GCGCGCCTGCAGCGTAGGCGCTAGGGACCTC-3’	

Bacterial growth

Pneumococci were grown on Columbia blood agar plates without antibiotics for 6 - 8 hours at 37 °C in an atmosphere of 5% CO_2. Afterwards bacteria were transferred to a fresh blood agar plate containing selective antibiotics and incubated for approximately 8 - 9 hours under the same growth conditions. Bacteria from the strains D39*Δcps* (WT), D39*ΔcpsΔstkP* and D39*ΔcpsΔphpP* were propagated into pre-warmed RPMI 1640 *modi* medium without L-glutamine, phenol red [GE Healthcare Bio-Sciences]. Therefore, RPMI medium was supplemented with 30.52 mM glucose, 2.05 mM glutamine, 0.65 mM uracil, 0.27 mM adenine, 1.1 mM glycine, 0.24 mM choline chloride, 1.7 mM $NaH_2PO_4 \cdot H_2O$, 3.8 mM Na_2HPO_4, and 27 mM $NaHCO_3$, 20 mM HEPES according to Schulz *et al*. 2014 (Schulz et al., 2014). All liquid cultures were inoculated to starting optical densities ($OD_{600\ nm}$) between 0.05 and 0.06 and growth was measured over 12h. Figure S1 displays the growth curves of WT, *ΔstkP* and *ΔphpP*.

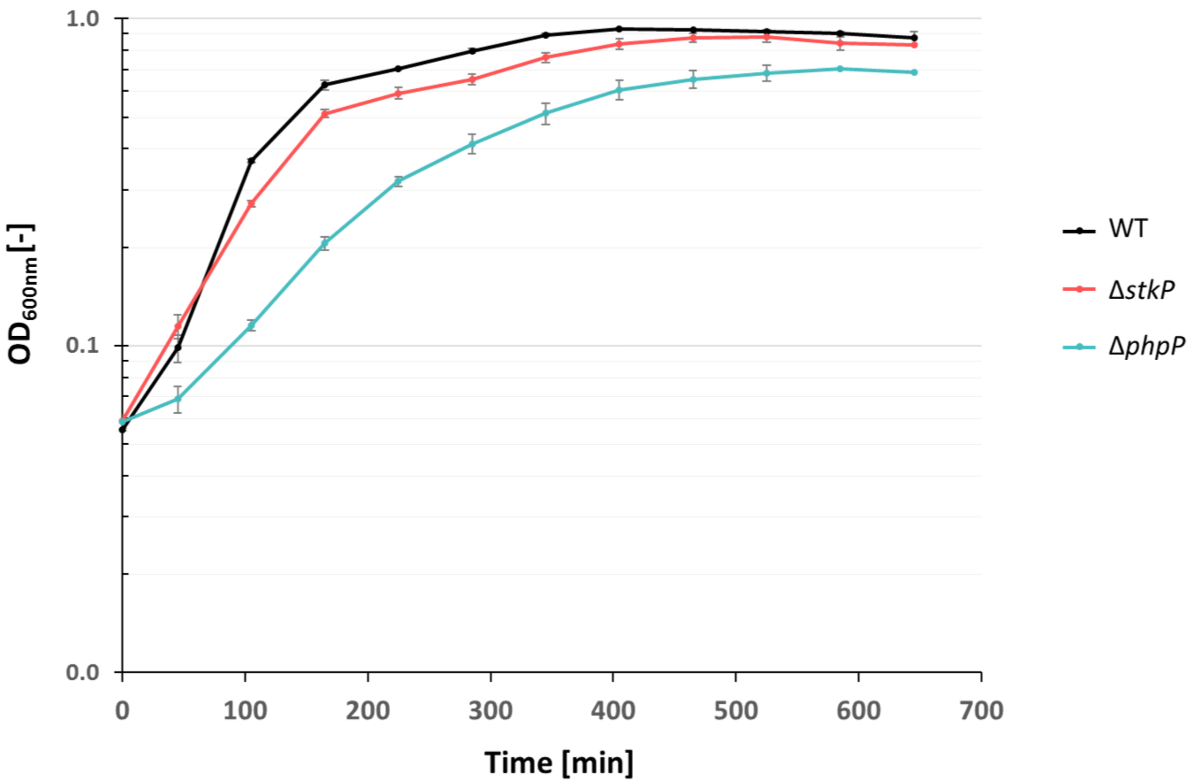

Fig.S1: Growth of *S. pneumoniae* in RPMi *modi* medium.

References

Chen, S., Songkumarn, P., Liu, J., and Wang, G.-L. (2009). A versatile zero background T-vector system for gene cloning and functional genomics. *Plant physiology* 150, 1111–1121. doi: 10.1104/pp.109.137125

Rennemeier, C., Hammerschmidt, S., Niemann, S., Inamura, S., Zähringer, U., and Kehrel, B. E. (2007). Thrombospondin-1 promotes cellular adherence of gram-positive pathogens via recognition of peptidoglycan. *FASEB journal : official publication of the Federation of American Societies for Experimental Biology* 21, 3118–3132. doi: 10.1096/fj.06-7992com

Saleh, M., Bartual, S. G., Abdullah, M. R., Jensch, I., Asmat, T. M., Petruschka, L., et al. (2013). Molecular architecture of *Streptococcus pneumoniae* surface thioredoxin-fold lipoproteins crucial for extracellular oxidative stress resistance and maintenance of virulence. *EMBO molecular medicine* 5, 1852–1870. doi: 10.1002/emmm.201202435

Schulz, C., Gierok, P., Petruschka, L., Lalk, M., Mäder, U., and Hammerschmidt, S. (2014). Regulation of the arginine deiminase system by ArgR2 interferes with arginine metabolism and fitness of *Streptococcus pneumoniae*. *mBio* 5. doi: 10.1128/mBio.01858-14

Tettelin, H., Nelson, K. E., Paulsen, I. T., Eisen, J. A., Read, T. D., Peterson, S., et al. (2001). Complete Genome Sequence of a Virulent Isolate of *Streptococcus pneumoniae*. *Science (New York, N.Y.)* 293, 498–506. doi: 10.1126/science.1059581
