# Supplementary material for: Proteomic Investigation Uncovers Potential Targets and Target Sites of Pneumococcal Serine-Threonine Kinase StkP and Phosphatase PhpP
Source: Front Microbiol. 2020 Feb 4;10:3101. doi: 10.3389/fmicb.2019.03101 (PMC7011611; doi:10.3389/fmicb.2019.03101)

Supplementary Image 1_A

Comparison of protein abundances Δ*phpP*/WT. Voronoi treemap of the quantified proteome (1,070 proteins of 1,918 theoretical proteins) of pneumococcal phosphatase mutant in comparison to WT assigned to specific functional main roles. Within the treemap all tiles symbolize single proteins which are colored according to their abundance in a divergent color gradient. Turquoise cells indicate proteins that are less abundant or “Off” in Δ*phpP* in comparison to the WT whereas higher abundant or “On” proteins are illustrated in orange. Grey fields represent proteins that could not be quantified in the compared strains.

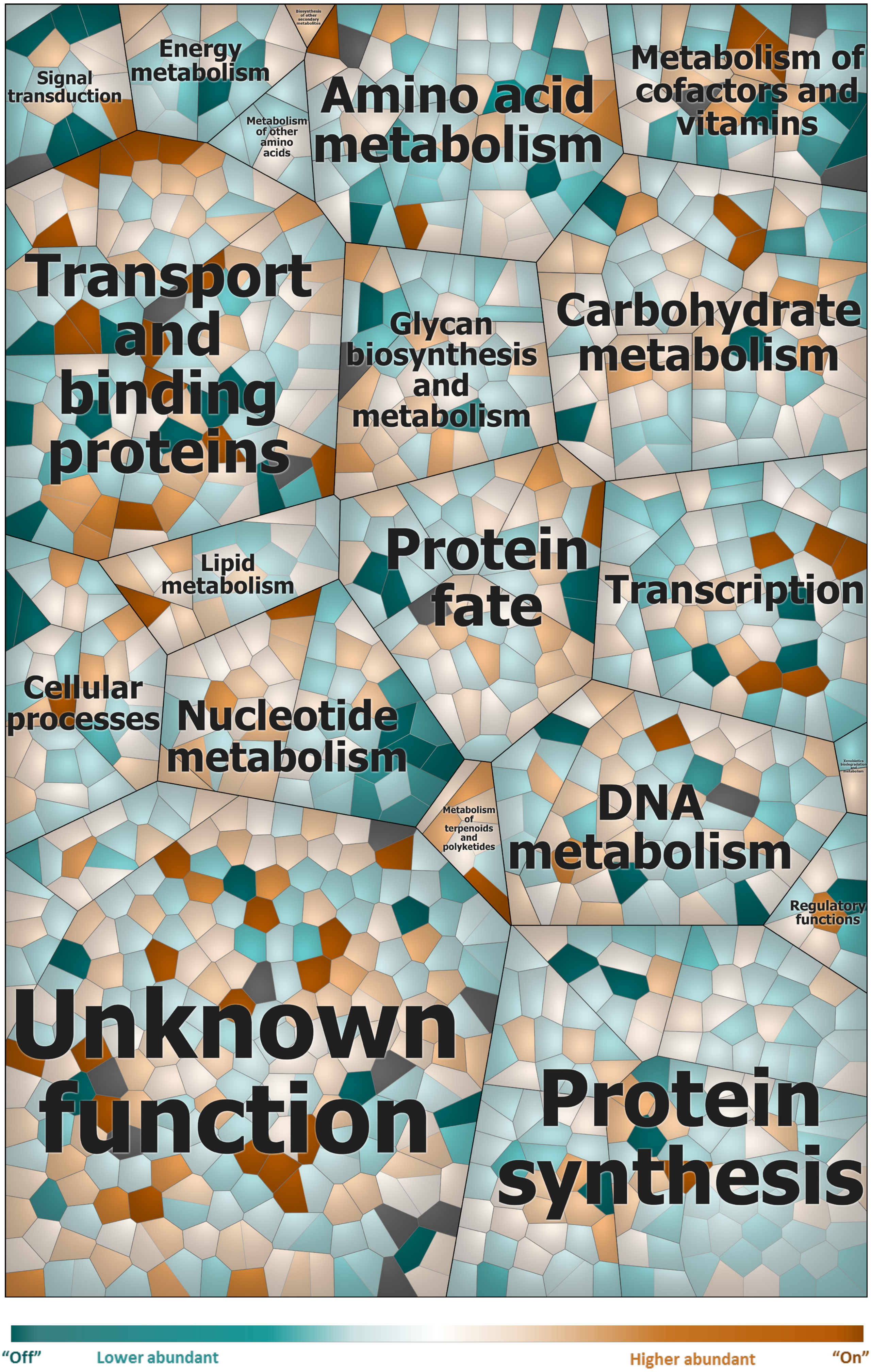

Supplementary Image 1_B

Comparison of protein abundances Δ*phpP*/WT. Voronoi treemap of the quantified proteome (1,070 proteins of 1,918 theoretical proteins) of pneumococcal phosphatase mutant in comparison to WT assigned to specific functional sub roles. Within the treemap all tiles symbolize single proteins which are colored according to their abundance in a divergent color gradient. Turquoise cells indicate proteins that are less abundant or "Off" in Δ*phpP* in comparison to the WT whereas higher abundant or "On" proteins are illustrated in orange. Grey fields represent proteins that could not be quantified in the compared strains.

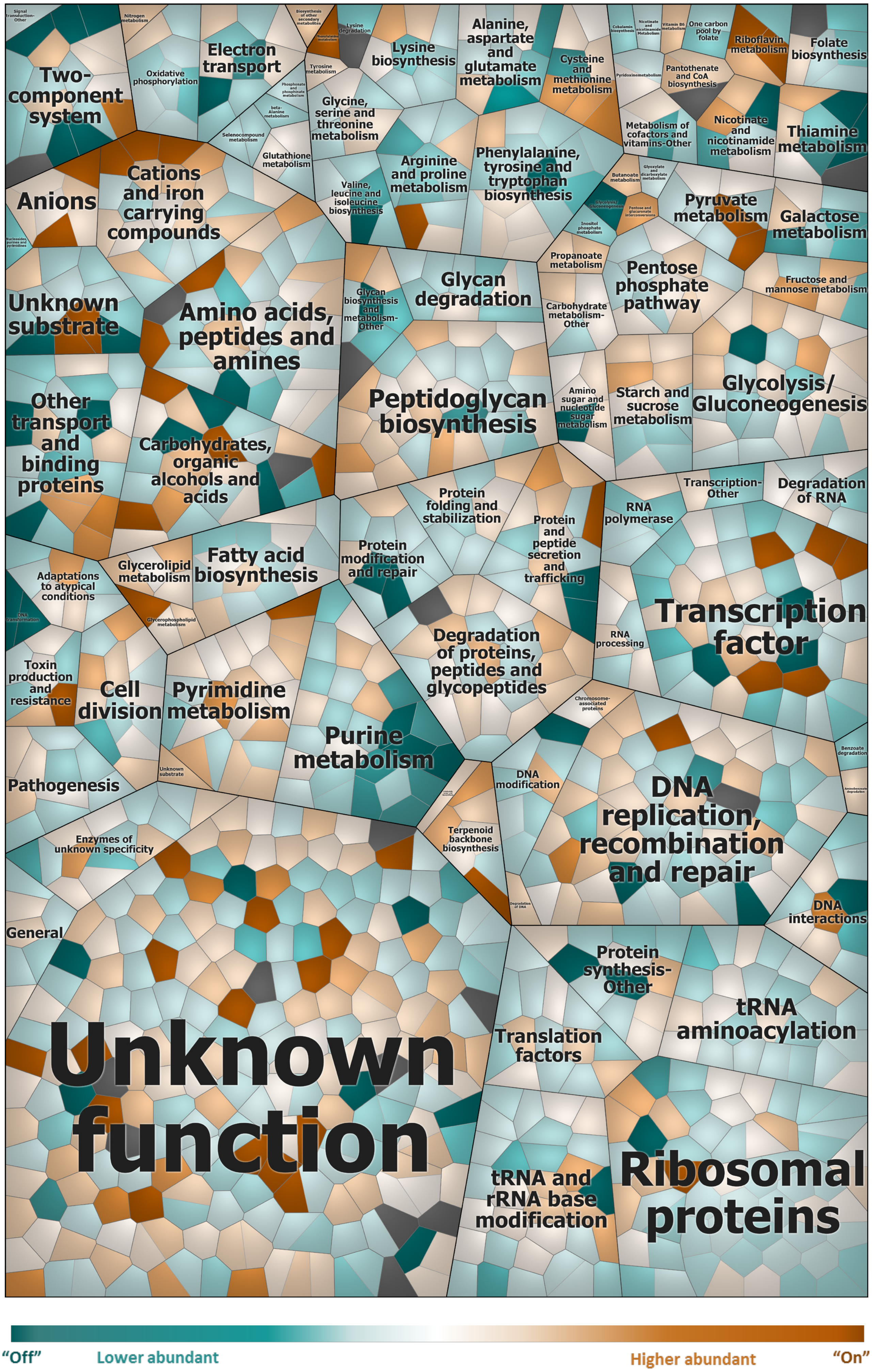

Supplementary Image 1_C

Comparison of protein abundances Δ*phpP*/WT. Voronoi treemap of the quantified proteome (1,070 proteins of 1,918 theoretical proteins) of pneumococcal phosphatase mutant in comparison to WT labelled with single proteins. Within the treemap all tiles symbolize single proteins which are colored according to their abundance in a divergent color gradient. Turquoise cells indicate proteins that are less abundant or "Off" in Δ*phpP* in comparison to the WT whereas higher abundant or "On" proteins are illustrated in orange. Grey fields represent proteins that could not be quantified in the compared strains.

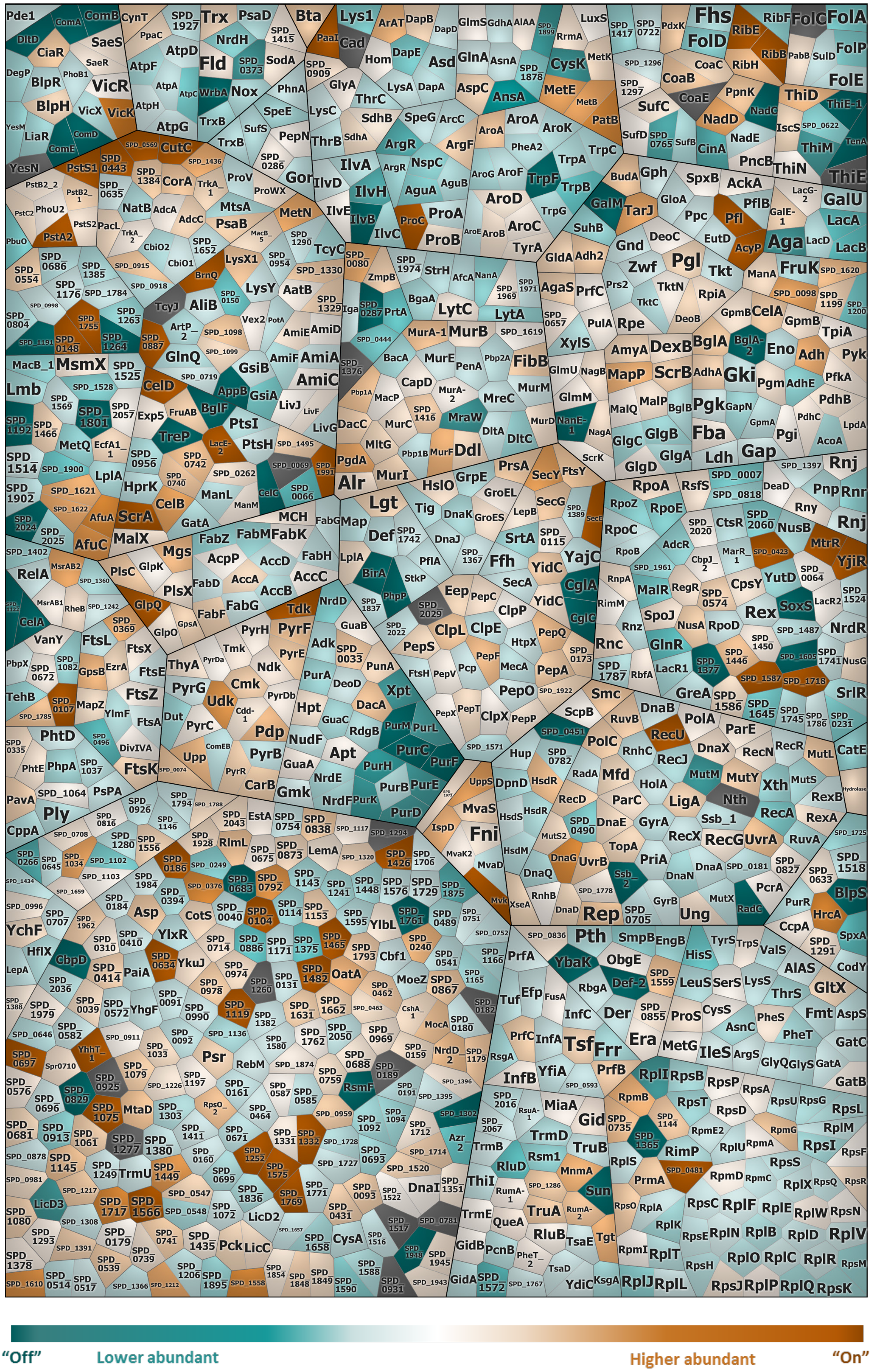

Supplementary Image 1_D

Comparison of protein abundances Δ*stkP*/WT. Voronoi treemap of the quantified proteome (1,070 proteins of 1,918 theoretical proteins) of pneumococcal kinase mutant in comparison to WT assigned to specific functional main roles. Within the treemap all tiles symbolize single proteins which are colored according to their abundance in a divergent color gradient. Turquoise cells indicate proteins that are less abundant or “Off” in Δ*stkP* in comparison to the WT whereas higher abundant or “On” proteins are illustrated in orange. Grey fields represent proteins that could not be quantified in the compared strains.

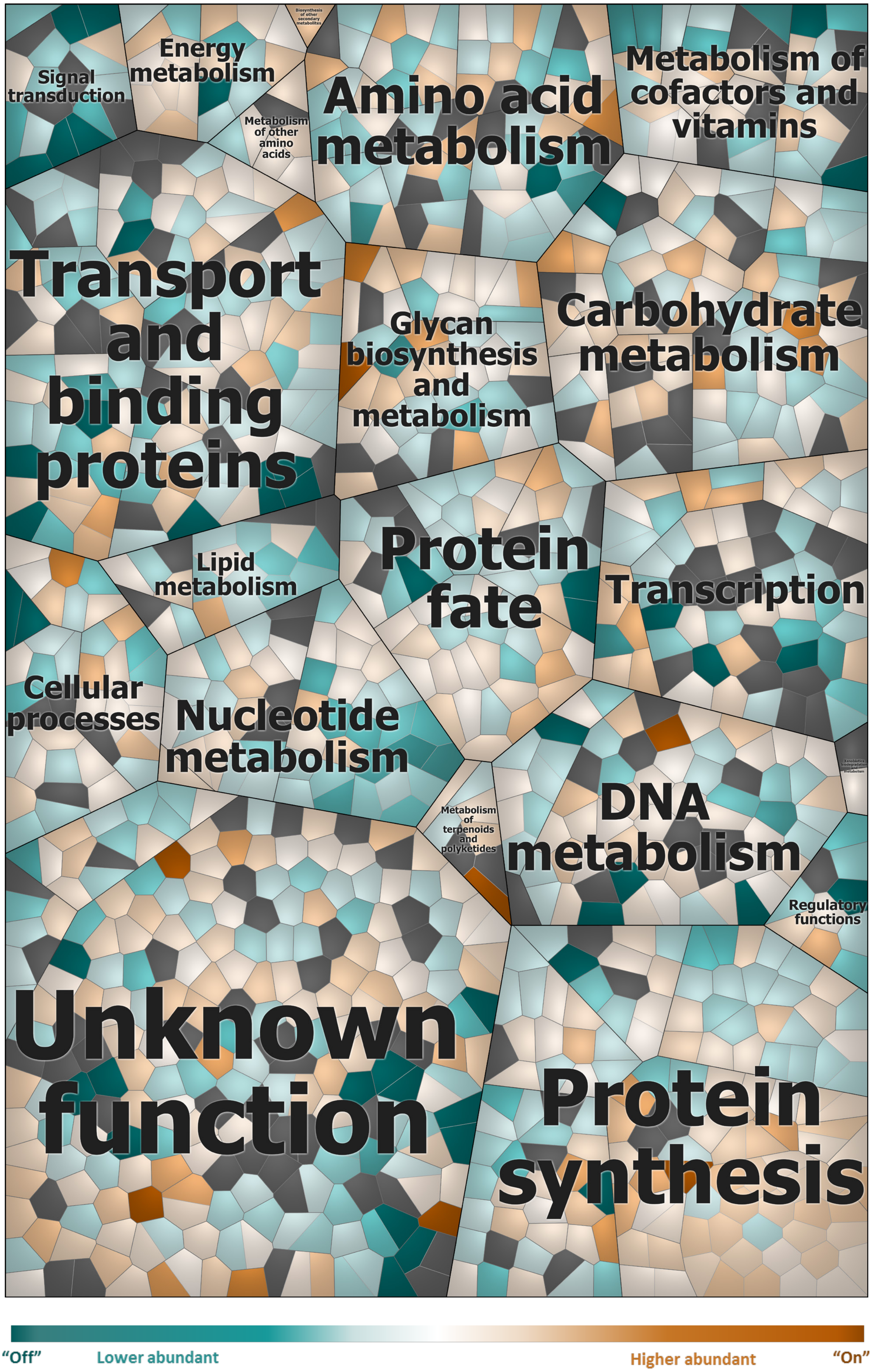

Supplementary Image 1_E

Comparison of protein abundances Δ*stkP*/WT. Voronoi treemap of the quantified proteome (1,070 proteins of 1,918 theoretical proteins) of pneumococcal kinase mutant in comparison to WT assigned to specific functional sub roles. Within the treemap all tiles symbolize single proteins which are colored according to their abundance in a divergent color gradient. Turquoise cells indicate proteins that are less abundant or “Off” in Δ*stkP* in comparison to the WT whereas higher abundant or “On” proteins are illustrated in orange. Grey fields represent proteins that could not be quantified in the compared strains.

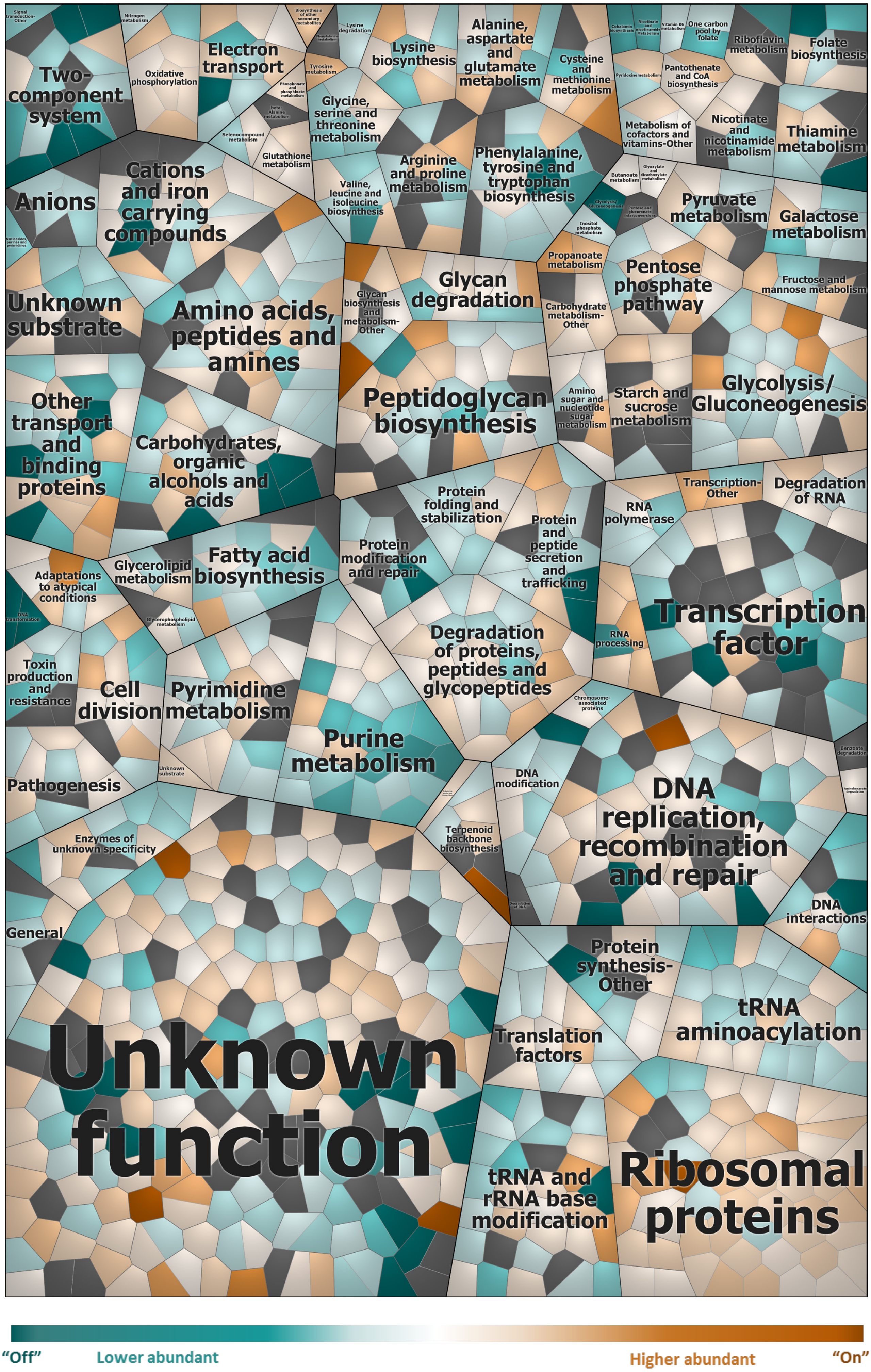

Supplementary Image 1_F

Comparison of protein abundances Δ*stkP*/WT. Voronoi treemap of the quantified proteome (1,070 proteins of 1,918 theoretical proteins) of pneumococcal kinase mutant in comparison to WT labelled with single proteins. Within the treemap all tiles symbolize single proteins which are colored according to their abundance in a divergent color gradient. Turquoise cells indicate proteins that are less abundant or "Off" in Δ*stkP* in comparison to the WT whereas higher abundant or "On" proteins are illustrated in orange. Grey fields represent proteins that could not be quantified in the compared strains.

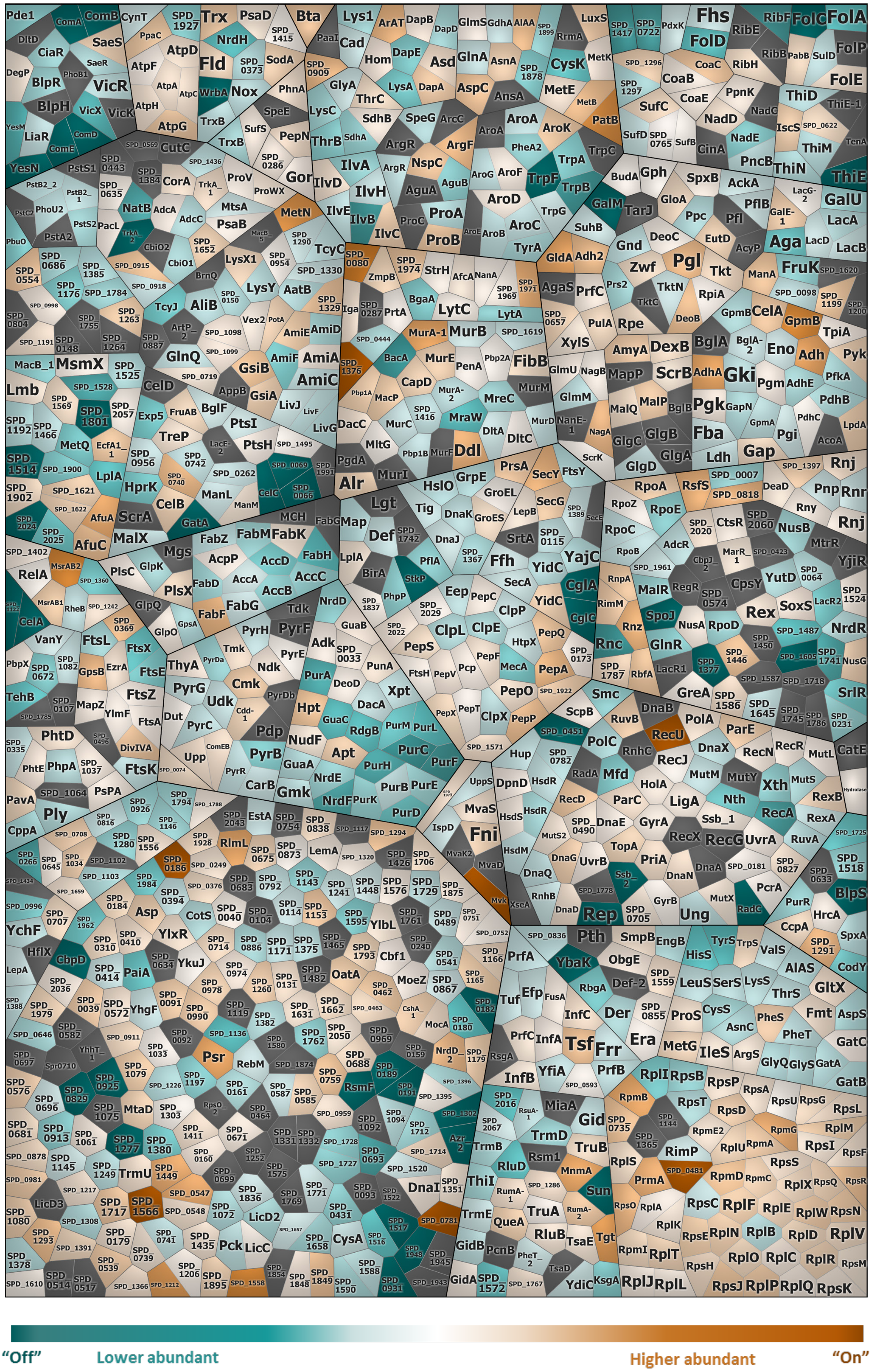

Supplement: Supplementary file 8 [file Image_1.pdf]
